# Insights of *β*‐Lactams Resistance in *Klebsiella pneumoniae* Clinical Isolates: A Focus on Molecular Identification of Drug Resistance

**DOI:** 10.1155/ijm/2707907

**Published:** 2026-01-29

**Authors:** Lavouisier F. B. Nogueira, Marília S. Maia, Marco A. F. Clementino, Ila F. N. Lima, Jorge L. N. Rodrigues, Luciana V. C. Fragoso, Glairta S. Costa, Jose Q. S. Filho, Alexandre Havt, Deiziane V. S. Costa, Lyvia M. V. C. Magalhães, Dilza Silva, José K. Sousa, Aldo A. M. Lima

**Affiliations:** ^1^ Institute of Biomedicine, Faculty of Medicine, Federal University of Ceará, Fortaleza, Ceará, Brazil, ufc.br; ^2^ Department of Medicine, Postgraduate Medical Microbiology Program, Federal University of Ceará, Fortaleza, Ceará, Brazil, ufc.br; ^3^ Hospital Universitário Walter Cantídio, Federal University of Ceará, Fortaleza, Ceará, Brazil, ufc.br; ^4^ Division of Infectious Diseases and International Health, University of Virginia, Charlottesville, Virginia, USA, virginia.edu; ^5^ Biomolecular Analysis Facility, School of Medicine, University of Virginia, Charlottesville, Virginia, USA, virginia.edu; ^6^ Department of Gastroenterology, Hepatology and Nutrition, Cincinnati Children′s Hospital Medical Center, Cincinnati, Ohio, USA, cincinnatichildrens.org

**Keywords:** *β*-lactamases, antimicrobial resistance, *Klebsiella pneumoniae*, molecular diagnosis, multidrug-resistant, qPCR

## Abstract

*Klebsiella pneumoniae* is associated with high antimicrobial resistance and is commonly isolated from colonization and healthcare‐associated infections (HAIs). This study is aimed at developing and validating molecular assays to detect resistance genes belonging to the *bla* family in resistant *K. pneumoniae* isolates. The genes included belong to the subfamilies: *bla*SHV*, bla*TEM*, bla*NDM*, bla*KPC*, bla*GES*, bla*CTX‐M, and relevant variants of the *bla*OXA subfamily. The identified genotypic profile showed a high prevalence of genes belonging to Ambler′s classes of beta‐lactamases A, B, and D, which was in accordance with the phenotypic results obtained for the isolates investigated. A high prevalence of resistance to penicillins, cephalosporins, and carbapenems was observed. In conclusion, the assays developed were efficient in detecting the main resistance genes of the *bla* family in *K. pneumoniae*, revealing a concerning regional burden of resistance genes.

## 1. Introduction

Bacterial resistance to antimicrobial agents is a global public health problem that leads to increased treatment costs, longer lengths of stay, and higher morbidity and mortality rates in hospitalized patients, especially in intensive care units (ICUs) [1, 2]. Therefore, understanding the dissemination of antimicrobial resistance (AMR) in the pathogenesis of bacterial colonization and/or infection can be of critical importance in preventing and controlling this issue.

*Klebsiella pneumoniae* is a gram‐negative *γ*‐proteobacterium, belonging to the *Enterobacteriaceae* family; it is generally viewed as an opportunistic microorganism, carrying several virulence factors and capable of accumulating resistance genes to various classes of antimicrobials. It is commonly associated with cases of colonization and/or healthcare‐associated infections (HAIs) and has been identified as an etiological agent in pneumonia, urinary tract infections (UTIs), soft tissue and surgical wound infections, bacteremia, and sepsis [3].

It is estimated that *K. pneumoniae* is responsible for approximately 10% of all HAIs, and of these, 32.8% are caused by strains resistant to multiple antimicrobial drugs. However, studies indicate that the rate of isolated strains exhibiting AMR has increased over the years [4, 5].

*K*. *pneumoniae* has been recognized as an emerging multidrug‐resistant (MDR) microorganism of emergency priority by the World Health Organization (WHO) for the development of new therapies. This designation stems from its association with the ability to overcome colonization resistance imposed by the gastrointestinal microbiota and its acquired nonsusceptibility to at least one agent in three or more antimicrobial categories, a defining characteristic of MDR organisms [6, 7]. Epidemiological data have demonstrated that *K. pneumoniae* can translocate from the gastrointestinal tract to other sterile sites of the same host or to other patients through the fecal–oral route, which highlights the clinical relevance of this microorganism [8].

One of the most likely causes of the increasingly frequent emergence of bacterial strains resistant to one or more antibiotics is the excessive and sometimes incorrect use of antimicrobials. The relatively long time required to identify the pathogen by traditional methods, as well as to obtain the results of the antimicrobial susceptibility test (AST) compels clinicians to use broad‐spectrum drugs empirically, thereby increasing selective pressure, which ultimately benefits pathogens genetically capable of adapting to the adverse environment [1, 9].

A possible solution to reduce the time needed to obtain a resistance profile is the development of molecular methodologies that can make resistance identification faster and also be more sensitive than traditional methodologies [9].

Beta‐lactams are the class of antimicrobials commonly affected by resistance, which is conferred in gram‐negative bacteria mainly by the *bla* gene family. This group of genes encodes enzymes called beta‐lactamases, which can neutralize the action of beta‐lactams through hydrolysis of the beta‐lactam ring [10–12].

In this study, we investigated the resistance phenotype and the genotypic profile of the *bla* family of *β*‐lactamases in *K. pneumoniae* strains isolated from patients admitted to the ICU in a university hospital in Fortaleza, Ceará, Brazil.

We aim to develop molecular assays capable of identifying all variants of the most relevant genes belonging to the *bla* family, specifically: *bla*SHV, *bla*TEM, *bla*NDM, *bla*KPC, *bla*GES, and *bla*CTX‐M. Additionally, the most epidemiologically relevant variants of the *bla*OXA subfamily were also included, thereby creating a set of primers capable of detecting hundreds of resistance gene variants with a reduced number of reactions.

## 2. Methods

### 2.1. Obtaining Bacterial Isolates and Identification

**Figure 1:**
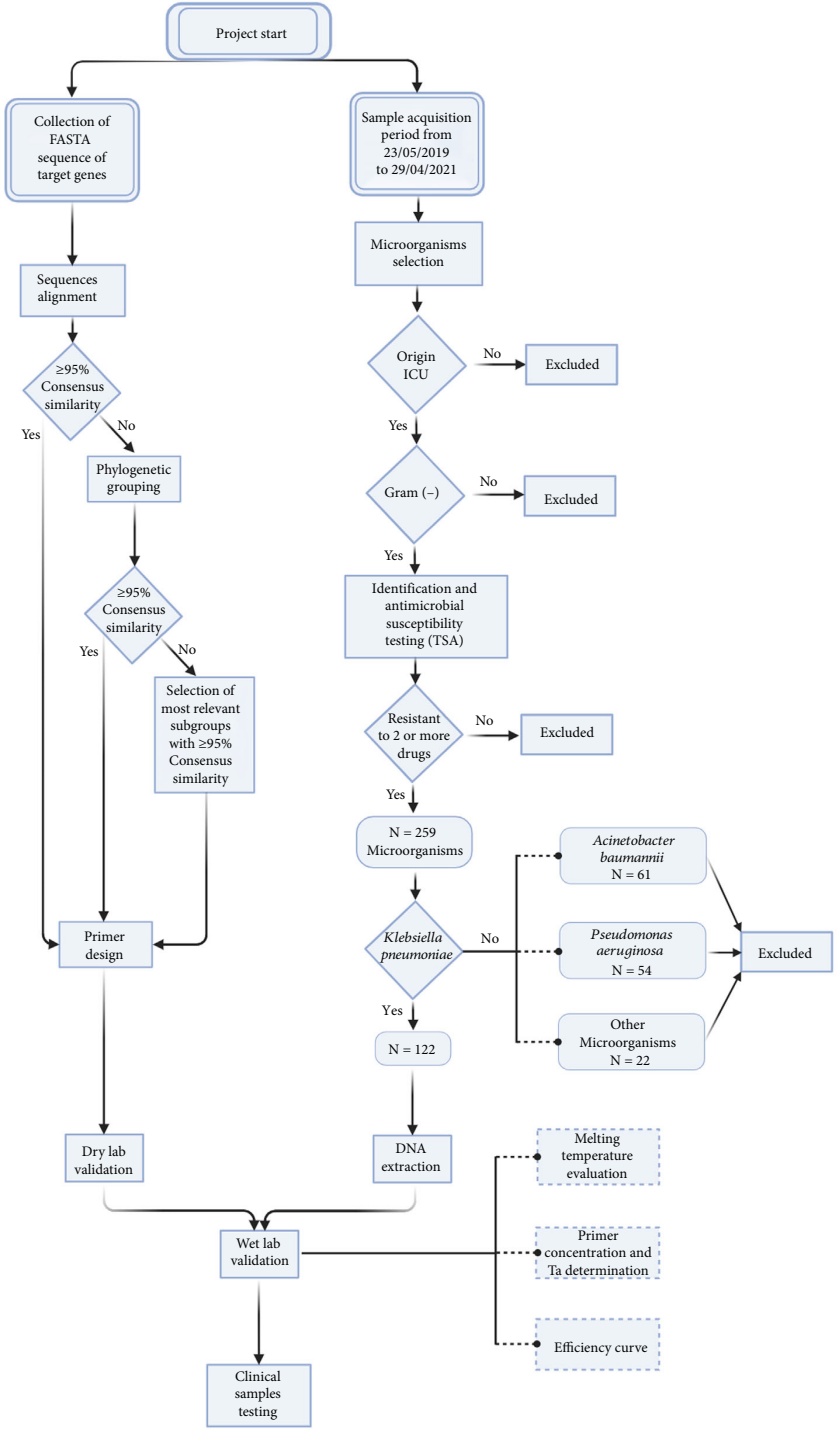
Flow chart outlining the comprehensive methodology of the project. Detailing the two main branches of the study: dry lab primer design and validation, and clinical lab sample acquisition and selection, culminating in wet lab validation and clinical samples testing. Date format: day/month/year. Created by using BioRender.

For this study, samples were collected as part of the clinical investigation of patients admitted to the ICU of a tertiary care health unit in Fortaleza, Ceará. The microorganisms included in this work were those identified as gram‐negative bacteria resistant to two or more groups of antimicrobial agents, including subclasses of *β*‐lactams, fluoroquinolones, and aminoglycosides (Figure 1).

The bacterial isolates were identified and tested for their susceptibility to antimicrobials using the automated VITEK 2 Compact method (BioMérieux, Marcy l′Etoile, France), according to the manufacturer′s recommendations. Minimum inhibitory concentrations were interpreted according to the Clinical and Laboratory Standards Institute (CLSI, M100‐30th ed., 2020). For quality control of susceptibility tests, strains from the American Type Culture Collection (ATCC) were used. Specimens with a resistance profile that met the research objectives were included in the study.

### 2.2. Extraction of Bacterial DNA

To extract the genetic material, the Wizard Genomic DNA Purification extraction and purification kit (Promega, Madison, United States) was used according to the manufacturer′s recommendations.

After extraction, all samples were then quantified by spectrophotometry using the NanoDrop 2000 (Thermo Fisher Scientific, Waltham, Massachusetts, United States) and stored in a −80°C freezer until used in the experiments.

### 2.3. Selection of Genes Used in the Study and Obtaining FASTA Sequences

For greater coverage of the genetic profile of *β*‐lactam resistance in gram‐negative bacteria, the most epidemiologically relevant genes belonging to the *bla* gene family were selected as part of the study. These included: *bla*SHV, *bla*TEM, *bla*NDM, *bla*KPC, *bla*GES, *bla*CTX‐M, and *bla*OXA [10, 13]. The sequences used were obtained from the Comprehensive Antibiotic Resistance Database (CARD) and National Center for Biotechnology Information (NCBI) platforms, which compile and organize the resistance gene sequences available in GenBank. A list of identifiable sequences is available in Text S1.

### 2.4. Primer Design

To design the primers, all variant sequences of each gene included in the study, available on the CARD and NCBI platforms, were gathered. The sequences were then aligned using Clustal Omega software (v1.2.2), and the alignments were analyzed using SnapGene software (v5.3.0) [14, 15].

Consensus regions with ≥ 95% homology were selected and used to design the primers using the Primer‐BLAST platform from the NCBI (United States). The consensus sequences obtained are available in Text S2 [16].

Genes with few conserved regions, for which it was not possible to obtain consensus sequences with ≥ 95% homology, were separated into clades. Primers were then designed for these sequences by phylogenetic grouping, ensuring a minimum of 95% similarity.

### 2.5. Dry Lab Validation of the Developed Primers

The dry lab validation of all developed primers was performed regarding their specificity, structure, and the formation of primer dimers and hairpins. The dry lab analyses were performed using the Primer‐BLAST (NCBI, United States) [17], and Sequence Manipulation Suite (SMS): PCR Primer Stats platforms [15].

### 2.6. Testing, Optimization, and Standardization of Primers

The reactions were standardized using in‐house–developed positive controls, which were obtained through the amplification of genetic material, isolation, and purification of amplicons originating from isolates phenotypically resistant to beta‐lactams. For the negative control, ultrapure water (DNase/RNase free) was used. Reactions were carried out using a SYBR Green master mix (Promega, Madison, United States). Initial results were evaluated based on the melting temperature (Tm) to confirm the specificity of the amplicons.

To determine the most efficient qPCR conditions, reduce nonspecificity, and facilitate result interpretation, a concentration gradient and annealing temperature (Ta) optimization of the primers were performed. The qPCR reaction conditions included a hot start step at 95°C for 2 min, followed by 35 cycles consisting of a denaturation step for 15 s at 95°C, and an annealing/extension step for 1 min at the Ta specific to each primer. All reactions concluded with a final melting curve step, with a temperature variation from 60°C to 95°C at an increase of 0.05°C/sec.

A 9‐point efficiency curve was performed for each developed primer, using a dilution factor of 1:8, with concentrations ranging from ≈27,438,596 to ≈2 copies/*μ*L. This procedure allowed us to determine the threshold values, evaluate the efficiency (%), the correlation coefficient (*R* ^2^), and the limit of detection for each primer [18, 19].

### 2.7. Detection of Resistance‐Related Genes by Molecular Biology

The *K. pneumoniae* isolates obtained in the study were tested against the developed primers. Melting curve analysis of all reactions was used to evaluate the specificity of the isolates′ results by comparison with the specific Tm of the positive control.

## 3. Results

### 3.1. Selection of Bacterial Samples

A total of 249 samples of gram‐negative bacteria resistant to beta‐lactam antimicrobials were collected from May 23, 2019, to April 29, 2021. Upon identification, it was verified that the most prevalent microorganism found was *K. pneumoniae*, accounting for 48.99% (122/249) of isolates, with 21.8% originating from infection cases and 78.2% from colonization cases.

### 3.2. Identification of the Phenotypic Profile of Beta‐Lactams Resistance of *K. pneumoniae* Strains

**Figure 2:**
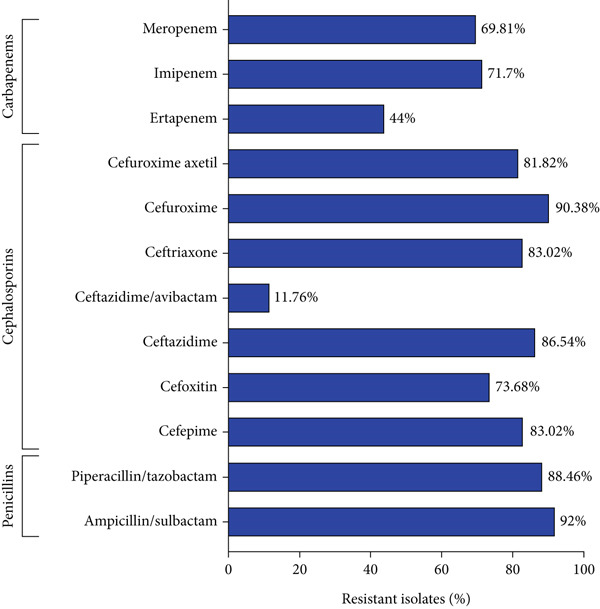
Prevalence of *K. pneumoniae* isolates resistant to the evaluated beta‐lactams (penicillins, cephalosporins, and carbapenems). This figure demonstrates a phenotypic resistance profile to multiple beta‐lactam drugs in approximately 70% of the isolates, with the exception of ceftazidime/avibactam and ertapenem.

The bacterial isolates included in the study were tested against a wide range of beta‐lactams drugs, and the presence of a high percentage of isolates resistant to these drugs was verified, which included penicillins: ampicillin/sulbactam (92.00%) and piperacillin/tazobactam (88.46%); cephalosporins: cefepime (83.02%), cefoxitin (73.68%), ceftazidime (86.54%), ceftazidime/avibactam (11.76%), ceftriaxone (83.02%), cefuroxime (90.38%), and cefuroxime axetil (81.82%), and carbapenems: ertapenem (44.00%), imipenem (71.70%), and meropenem (69.81%), as shown in Figure 2.

### 3.3. Primers Design

After selection from the CARD and NCBI (United States) platforms, and subsequent compilation and analysis of FASTA sequences, it was possible to identify consensus sequences common to all variant sequences available in the databases for each of the genes included in the study: *bla*SHV (*N* = 156), *bla*TEM (*N* = 167), *bla*NDM (*N* = 27), *bla*KPC (*N* = 208), *bla*GES (*N* = 25), and *bla*CTX‐M (*N* = 144). The *bla*OXA gene was an exception, for which the most clinically relevant sequences were used (*N* = 203).

The *bla*CTX‐M and *bla*OXA genes, due to their diversity and high degree of genetic variation between homologous sequences, were grouped into clades and further divided into subgroups. The *bla*CTX‐M gene group was named according to international standardization for this gene, with the *bla*CTX‐M‐1‐like variants being divided into two groups due to their high number of sequences and genetic variation.

**Figure 3:**
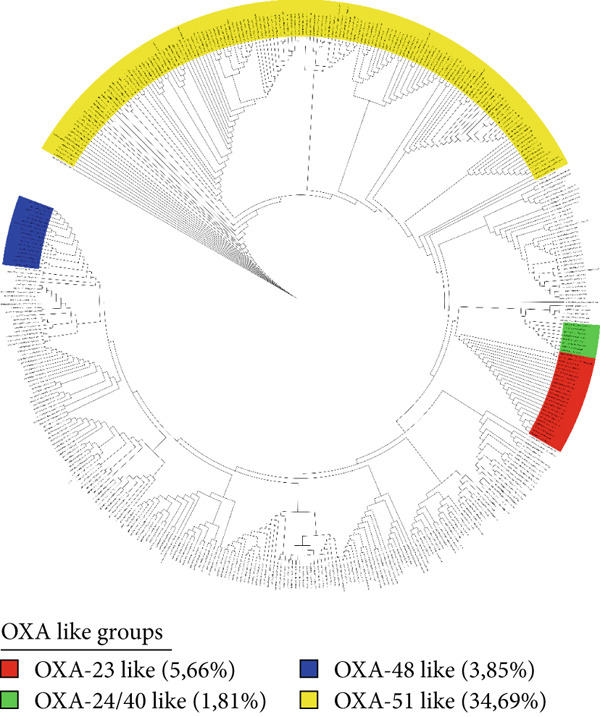
Cladogram of the *bla*OXA subfamily, showing the most epidemiologically relevant groups covered by the developed primers. Also shown is the percentage of sequences identifiable by each primer in comparison with the total number of the *bla*OXA family (*N* = 441) at the time of this work′s publication.

**Figure 4:**
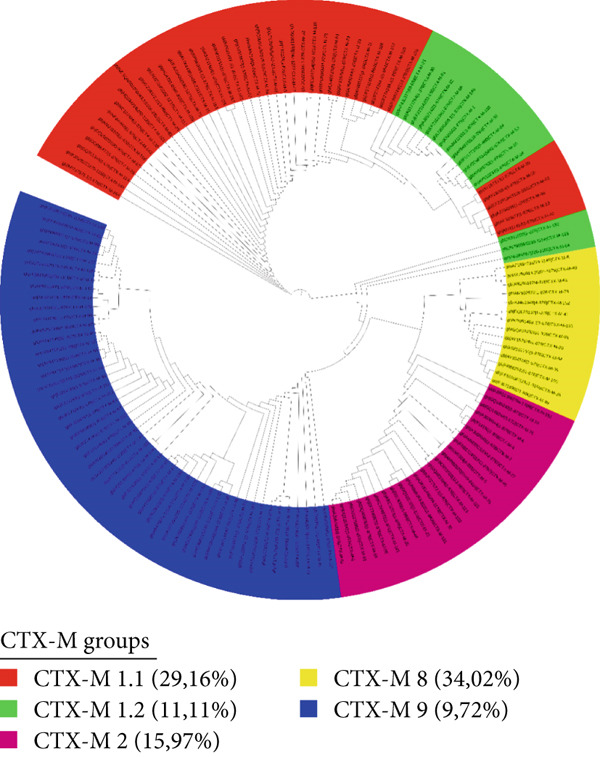
Cladogram showing all sequences from the *bla*CTX‐M subfamily divided into groups, illustrating the identifiable sequences and the percentage of sequences detectable by each primer. This is presented in comparison with the total number of the *bla*CTX‐M family (*N* = 144) up to the time of this work′s publication.

The group organization for *bla*CTX‐M and *bla*OXA genes was defined as follows: CTX‐M 1.1*like* (*N* = 42), CTX‐M 1.2*like* (*N* = 16), CTX‐M 2*like* (*N* = 23), CTX‐M 8*like* (*N* = 14), and, CTX‐M 9*like* (*N* = 49). The *bla*OXA gene, meanwhile, was divided into OXA*-23like* (*N* = 25), OXA*-24/40like* (*N* = 8), OXA*-48like* (*N* = 17), and OXA*-51like* (*N* = 153), as shown in Figures 3 and 4.

A total of 14 primer pairs were developed, which together have the capacity to detect 929 variants of resistance genes belonging to the *bla* gene family. The primer sequences for genes with conserved and nonconserved regions can be found in Tables 1 and 2, respectively.

**Table 1:** Primer sequences for conserved genes.

**Name of gene**	**Number of detectable variants**	**Primer name**	**Sequence**	**Primer size**
*bla*SHV	156	SHV‐F	ATTATCTCCCTGTTAGCCACCC	22
		SHV‐R	GTTTAATTTGCTCAAGCGGCTG	22
*bla*TEM	167	TEM‐F	ACCCAGAAACGCTGGTGAAA	20
		TEM‐R	GGGGCGAAAACTCTCAAGGA	20
*bla*NDM	27	NDM‐F	GAAGCTGAGCACCGCATTAG	20
		NDM‐R	CCATTTGCTGGCCAATCGTC	20
*bla*KPC	208	KPC‐F	TCGCGGAACCATTCGCTAAA	20
		KPC‐R	GAATGAGCTGCACAGTGGGA	20
*bla*GES	25	GES‐F	GCCCAGGAGAGAGATTACGC	20
		GES‐R	CTTGACCGACAGAGGCAACT	20

**Table 2:** Primer sequences for nonconserved genes divided into clades.

**Name of gene**		**Number of detectable variants**	**Primer name**	**Sequence**	**Primer size**
*bla*CTX‐M	*bla*CTX‐M 1.1*like*	42	CTXM1.1L‐F	GATTGCGGAAAAGCACGTCA	20
			CTXM1.1L‐R	TTCATCGCCACGTTATCGCT	20
	*bla*CTX‐M 1.2*like*	16	CTXM1.2L‐F	CGCCGCTGATTCTGGTCA	18
			CTXM1.2L‐R	TGACGATTTTAGCCGCCGAC	20
	*bla*CTX‐M 2*like*	23	CTXM2L‐F	ATGGCGCAGACCCTGAAAAA	20
			CTXM2L‐R	CTGCCGGTTTTATCGCCCA	19
	*bla*CTX‐M 8*like*	14	CTXM8L‐F	CGCTCAACACCGCGATCC	18
			CTXM8L‐R	ATCCCCGACAACCCACGAT	19
	*bla*CTX‐M 9*like*	49	CTXM9L‐F	CGTGGCTCAAAGGCAATACG	20
			CTXM9L‐R	TCTGTTGCGGCTGGGTAAAA	20

*bla*OXA	*bla*OXA‐23*like*	24	OXA23L‐F	GCTCTAAGCCGCGCAAATAC	20
			OXA23L‐R	TGACCTTTTCTCGCCCTTCC	20
	*bla*OXA‐24/40*like*	8	OXA24/40L‐F	TGCCGATGACCTTGCACATA	20
			OXA24/40L‐R	CCATTAGCTTGCTCCACCCA	20
	*bla*OXA‐48*like*	17	OXA48L‐F	CGGTAGCAAAGGAATGGCAAG	21
			OXA48L‐R	GGGCGATCAAGCTATTGGGA	20
	*bla*OXA‐51*like*	153	OXA51L‐F	GATCGGCCTTGAGCACCATA	20
			OXA51L‐R	GCCATAACCAACACGCTTCA	20

### 3.4. Dry Lab Primers Validation

The parameters obtained for each primer after analysis using Primer‐BLAST (NCBI, United States) and SMS PCR Primer Stats software (v2.0), are available in Table 3.

**Table 3:** Parameters obtained during dry lab validation of the developed primers.

**Gene**	**Primer name**	**Tm (C°)**	**GC%**	**Self-complementarity**	**Self-3** ^′^ **-complementarity**	**Product length**
*bla*SHV	SHV‐F	59.2	50.0	3	0	70
	SHV‐R	59.3	45.4	5	3	
*bla*TEM	TEM‐F	60.1	50.0	4	0	110
	TEM‐R	59.9	55.0	2	1	
*bla*NDM	NDM‐F	59.3	55.0	5	1	86
	NDM‐R	60.1	55.0	6	2	
*bla*KPC	KPC‐F	60.3	50.0	4	2	128
	KPC‐R	60.3	55.0	5	1	
*bla*GES	GES‐F	59.9	60.0	3	2	94
	GES‐R	59.9	55.0	3	2	
*bla*CTX‐M 1.1*like*	CTXM1.1L‐F	59.7	50.0	4	1	87
	CTXM1.1L‐R	60.1	50.0	4	2	
*bla*CTX‐M 1.2*like*	CTXM1.2L‐F	60.1	61.1	3	2	90
	CTXM1.2L‐R	61.0	55.0	3	3	
*bla*CTX‐M 2*like*	CTXM2L‐F	60.5	50.0	4	0	155
	CTXM2L‐R	60.7	57.8	4	1	
*bla*CTX‐M 8*like*	CTXM8L‐F	61.5	66.6	4	2	197
	CTXM8L‐R	60.9	57.8	3	2	
*bla*CTX‐M 9*like*	CTXM9L‐F	59.9	55.0	3	2	180
	CTXM9L‐R	60.1	50.0	3	0	
*bla*OXA‐23*like*	OXA23L‐F	60.0	55.0	4	0	129
	OXA23L‐R	59.9	55.0	2	0	
*bla*OXA‐24/40*like*	OXA24/40L‐F	59.7	50.0	4	2	177
	OXA24/40L‐R	60.0	55.0	4	0	
*bla*OXA‐48*like*	OXA48L‐F	59.8	52.3	3	0	183
	OXA48L‐R	59.8	55.0	4	0	
*bla*OXA‐51*like*	OXA51L‐F	59.8	55.0	4	2	199
	OXA51L‐R	59.1	50.0	2	1	

### 3.5. Wet Lab Testing, Optimization, and Standardization of Reactions

All primers were tested using in‐house–developed positive controls. It was verified that the ideal Ta for the developed primer pairs was 61°C, except for the primers targeting the *bla*NDM gene, for which the ideal Ta was 64°C. The reactions were subsequently evaluated for their specificity and stability by analyzing the Tm. The melting curves are available in Figure S1.

### 3.6. Efficiency Curve

It was found that all tested primers exhibited an efficiency rate between ≥ 93.21% and ≤ 101.28%, an *R* ^2^ ≥ 0.99, and a detection limit between ≈2 and ≈13 copies/*μ*L, as shown in Table 4.

**Table 4:** Results of the efficiency curves of the developed primers.

**Gene name**	**Efficiency (%)**	**Correlation coefficient (** **R** ^2^ **)**	**Threshold**	**Detection limit (copies/*μ*L)**	**Melting peak (Tm °C)**
*bla*SHV	96.15	0.992	0.60	≈2	86.7
*bla*TEM	95.32	1.000	0.70	≈2	81.5
*bla*NDM	99.90	0.999	0.48	≈2	85.7
*bla*KPC	100.44	0.992	0.24	≈2	85.7
*bla*GES	100.21	0.997	0.27	≈2	81.7
*bla*CTX‐M 1.1*like*	95.75	0.999	0.40	≈13	84.3
*bla*CTX‐M 1.2*like*	100.82	0.994	0.32	≈13	83.2
*bla*CTX‐M 2*like*	98.09	0.998	0.30	≈2	86.4
*bla*CTX‐M 8*like*	95.32	1.000	0.10	≈13	88.4
*bla*CTX‐M 9*like*	97.82	0.996	0.10	≈2	89.0
*bla*OXA‐23*like*	99.51	1.000	0.15	≈2	78.4
*bla*OXA‐24/40*like*	101.82	0.988	0.15	≈13	78.7
*bla*OXA‐48*like*	93.21	1.000	0.20	≈13	80.29
*bla*OXA‐51*like*	95.51	0.993	0.55	≈2	82.0

### 3.7. Identification of the Genetic Resistance Profile of *K. pneumoniae* Isolates

The 122 K. pneumoniae isolates were tested against the 14 developed primer pairs, revealing a high prevalence of beta‐lactam resistance genes among the analyzed isolates. The most prevalent genes detected were: blaKPC (95.90%); blaSHV (94.26%); blaCTX‐M 1.2like (88.52%); blaCTX‐M 2like (83.61%); blaCTX‐M 1.1like (80.33%); blaTEM (80.33%); blaNDM (33.61%); blaOXA‐23like (28.69%); blaGES (20.49%); blaOXA‐51like (15.57%); blaCTX‐M 9like (13.93%); blaCTX‐M 8like (12.30%); blaOXA‐24/40like (11.48%); and blaOXA‐48like (3.28%). These results are shown in Figure 5a, grouped according to Ambler′s classification.

Figure 5(a) Prevalence of beta‐lactam resistance genes among the analyzed isolates, grouped according to the Ambler′s classification. (b) Percentage of *K. pneumoniae* isolates showing accumulation of genes encoding beta‐lactamases belonging to the *bla* family.

**Figure:**
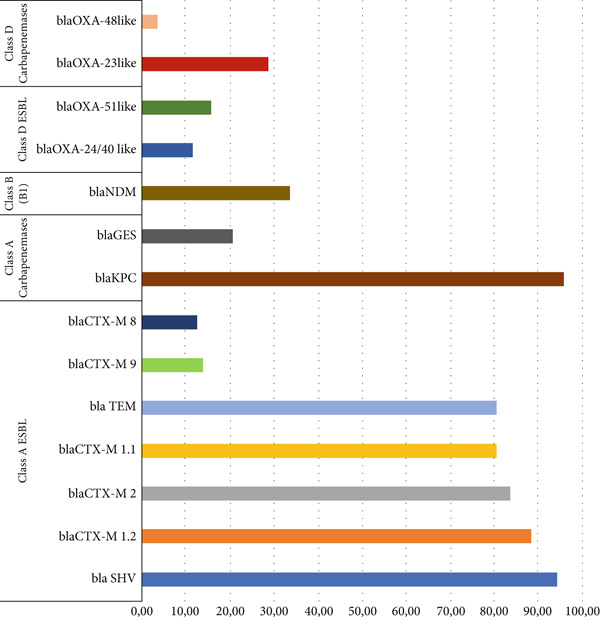
(a)

**Figure:**
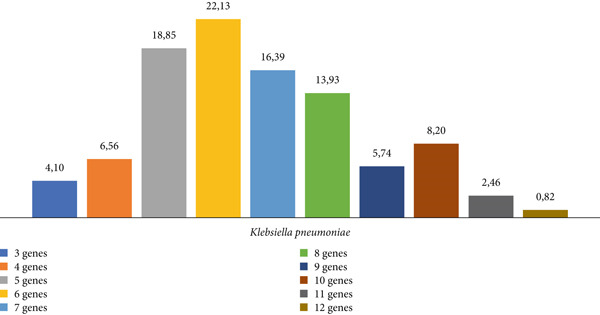
(b)

It is important to note that not all variants of each gene group analyzed belong to a single class in Ambler′s classification. For example, not all blaTEM and blaSHV gene variants exhibit activity as extended‐spectrum beta‐lactamases (ESBLs). However, due to the grouping of all sequences into a single primer, separating these sequences into other groups would be impossible. Therefore, the grouping of genes was performed comprehensively to cover the main classification of the respective subfamily.

Through the data obtained from the developed qPCR reactions, it was also possible to verify that the isolates analyzed during this study exhibited the accumulation of several genes belonging to the *bla* family. The number of tested genes present per isolate ranged from 3 to 12, as shown in Figure 5b.

## 4. Discussion

During the period analyzed in the study, 249 bacterial isolates resistant to two or more classes of antimicrobials used in TSA were identified. Of these resistant isolates, 48.99% (122/249) belonged to the species *K. pneumoniae*, which was the most prevalent microorganism among those identified.

These data are consistent with observations in Egypt, where when evaluating 186 samples from Cairo hospitals, *K. pneumoniae* was found as the main gram‐negative bacterial agent, representing 40.9% (76/186) of the identified microorganisms. Among these obtained isolates, a high prevalence of resistance to beta‐lactams, quinolones, and sulfonamides was identified (89.4%, 89.4%, and 87.1%, respectively) [20].

The phenotypic profile of the *K. pneumoniae* isolates analyzed in the present study demonstrated high levels of resistance to the beta‐lactam drugs tested across all evaluated classes (penicillins, cephalosporins, and carbapenems). Although the resistance profile of strains may vary depending on the testing location and conditions to prevent spread, beta‐lactam resistance among *Enterobacteriaceae* is known to be high, as demonstrated in a study conducted in Brazil with samples of human and veterinary origin, where 62.85% and 54.28% of isolated *Enterobacteriaceae* were resistant to amoxicillin/clavulanate and cefazolin, respectively [21].

In studies conducted in Egypt, evaluating *K. pneumoniae* isolates from food sources, and in Tunisia and Iran, evaluating isolates from hospital samples, high resistance to beta‐lactams was verified for ampicillin/sulbactam (93%), ceftazidime (95.5%), cefoxitin (95.5%), cefotaxime (93.2%), amoxicillin/clavulanate (86.4%), ertapenem (90.9%), and temocillin (84.0%) [22–24].

Likewise, in a study conducted with 102 isolates obtained from two Portuguese hospitals, resistance rates > 90% were verified for all beta‐lactam drugs tested, except for cephalosporins (cefoxitin: 40.2%; cefotetan: 68.6%) and carbapenems (ertapenem: 23.5%; imipenem: 32.4%; meropenem: 34.3%; and doripenem: 33.3%) [25].

This result is consistent with that found in our study, where the beta‐lactam drugs that showed a lower resistance rate belonged to the same classes mentioned, namely: cefoxitin (73.68%), ceftazidime/avibactam (11.76%), ertapenem (44.00%), imipenem (71.70%), and meropenem (69.81%).

The high prevalence of *K. pneumoniae* and the high rates of AMR verified in this work and in several papers for this microorganism align with its classifications as a severe threat to public health, being recognized as one of the Top 6 most fatal bacterial causative agents associated with AMR. The emergence and proliferation of MDR strains is inextricably linked to complex microbial factors such as mutations and horizontal genetic transfer [26].

The beta‐lactam resistance is conferred in gram‐negative bacteria mainly by genes belonging to the *bla* family. This group of genes is composed of dozens of subfamilies and hundreds of genetic subvariants. The developed molecular panel managed to encompass some of the most predominant and clinically relevant subfamilies among clinical isolates, as identified in several studies, namely: *bla*SHV, *bla*TEM, *bla*NDM, *bla*KPC, *bla*GES, *bla*CTX‐M, and *bla*OXA [27–29].

After analysis using Primer‐BLAST (NCBI, United States) and Primer Stats SMS software, the developed primers were found to exhibit specificity for the sequences used in the alignment, Tms between 59.29°C and 61.55°C, and GC concentrations varying between 45.45% and 66.67%. Additionally, they showed complementary base numbers ≤ 6 in the analysis of dimer and hairpin formation. The values obtained for these parameters are therefore compatible with those reported in reference studies [30, 31].

Due to the high number of sequences included in the development of primers, many of the alternatives obtained through Primer‐BLAST (NCBI, United States) did not meet the stability requirements regarding the formation of secondary structures (self‐annealing and hairpins). To select options with the best performance for these parameters, the GC clamp was relaxed. Consequently, primers SHV‐F, SHV‐R, CTXM1.2L‐R, and CTXM2L‐R obtained four G or C residues within the last five nucleotides of the 3 ^′^ end, whereas primers CTXM9L‐R and CTXM2L‐F did not exhibit C or G residues within the last five nucleotides of their sequences.

This could potentially lead to the formation of strong bonds and an increase in Tm for primers with more than three terminal GC residues, or cause amplification inhibition due to weak bonds when terminal CG residues are absent [32, 33]. Such changes, however, were not evident during wet lab primer testing.

The variety of sequences detectable by the developed primers makes wet lab validation with positive controls for all sequences virtually impossible. Dry lab analysis enabled the quick, cost‐effective, and continuous evaluation of all tested sequences, as the developed primers can be used to test against new variants of the target genes [30, 31, 34].

Wet lab analyses were conducted using the SYBR Green master mix. The melting curve was therefore employed as a parameter to evaluate reaction specificity. It was verified that for all tested primers, a single melting peak was observed, indicating the formation of a single amplicon and excluding the presence of secondary structures (primer dimers and hairpins).

Analysis of the melting curve and Tm is considered fundamental for testing the specificity of reactions that utilize SYBR Green as an amplification indicator. Unlike reactions employing specific probes, which emit fluorescence only when the reaction targets the expected sequence, SYBR Green functions by binding to any formed double‐stranded fragment, thereby emitting fluorescence. Consequently, only by analyzing the melting curve and the amplicon′s specific Tm is it possible to determine whether the reaction was successful [33, 35, 36].

The efficiency curve and its parameters were utilized to evaluate the performance of qPCR reactions by assessing how efficiently targets were amplified in each PCR cycle [32, 34]. Upon evaluating primer activity in qPCR reactions using the efficiency curve, all primers analyzed in this study demonstrated efficiency values of > 90% and < 110% and correlation coefficients (*R* ^2^) > 0.9, consistent with parameters outlined in reference literature [19, 37]. Furthermore, a high‐detection capacity was observed even at low concentrations of genetic material, as the obtained detection limit varied between ≈2 and ≈13 copies/*μ*L of the target gene used as a control.

Several studies report a growing prevalence of the *bla*KPC gene, identifying it as the most frequently detected carbapenemase worldwide. Prevalence values vary geographically. The main variant detected is *bla*KPC‐2, with prevalence rates ranging from 1.2% to 51.6% among *Enterobacteriaceae* in studies conducted in the United States and China, respectively [33, 35]. This gene is more frequently found in *K. pneumoniae* isolates, with many studies reporting high‐prevalence rates in this species, ranging from 17.2% to 64.6% [4, 38–40]. In a study conducted in an ICU in the northeast region of Brazil, 100% of 25 *K. pneumoniae* isolates obtained during the research exhibited the *bla*KPC gene [41].

The *bla*KPC gene values reported in this study may reflect the fact that the developed primer detects all gene variants as well as the higher resistance rates typically found in ICUs [42]. These results align with other studies performed in Northeast Brazil [37], which may indicate a concerning presence of the *bla*KPC gene among *K. pneumoniae* isolates in this Brazilian region.

The *bla*TEM and *bla*SHV genes were the first ESBLs identified and, along with the *bla*CTX‐M subfamily, are considered the most prevalent ESBLs. These genes are widely distributed among *Enterobacteriaceae*. In a study conducted in Sudan, the *bla*TEM, *bla*CTX‐M, and *bla*SHV genes were identified in 86.0%, 78.0%, and 28.0% of isolates, respectively [43].

A study conducted in ICUs in Chile reported the presence of these genes with prevalence rates of 81.0%, 84.7%, and 73.0% for *bla*SHV, *bla*CTX‐M‐1, and *bla*TEM, respectively. A higher prevalence of the *bla*SHV gene was observed compared with the study conducted in Sudan [44].

In Brazil, high prevalence rates of these genes have also been observed among *K. pneumoniae* isolates from ICUs, with studies indicating rates of 100%, 96%, and 72% for *bla*TEM, *bla*SHV, and *bla*CTX‐M1, respectively [40]. These results align with those found in the present study for the *K. pneumoniae* isolates tested.

The *bla*NDM gene is considered the second most prevalent carbapenemase‐producing gene globally, surpassed only by the *bla*KPC gene. In this study, the metallo‐beta‐lactamase *bla*NDM gene was detected in 33.61% of the isolates. Similar results were observed in China, where the gene was identified in 35.7% of 935 carbapenem‐resistant *Enterobacteriaceae* tested [45, 46, 47, 49].

Regarding oxacillinases, a low prevalence of the *bla*OXA‐48‐like gene was observed (3.28%). Although this gene can be present in all *Enterobacteriaceae*, it is more prevalent in *K. pneumoniae* strains [20]. Literature values for the prevalence of this gene in *Enterobacteriaceae* are highly divergent, showing significant variations related to the study location, with rates ranging from 7.3% in China to 83.3% in Morocco [40, 48]. In a Brazilian study that analyzed 4451 *Enterobacteriaceae* isolates, the *bla*OXA‐48‐like gene was detected in only 2.5% of the tested isolates [49]. Thus, the result obtained in this study is compatible with what was previously identified in Brazil and is similar to the findings in China.

The presence of *bla*OXA gene variants (*bla*OXA‐51‐like [15.57%], *bla*OXA‐24/40‐like [11.48%], and *bla*OXA‐23‐like [28.69%]) was also observed in the tested isolates. These genes were long considered exclusive to *Acinetobacter baumannii* strains; however, studies have demonstrated their presence in other *Enterobacteriaceae*, including *K. pneumoniae* [50]. Results regarding the prevalence of these genes in *K. pneumoniae* strains remain scarce. Nevertheless, in a study conducted in Bahrain (Persian Gulf), the *bla*OXA‐51 and *bla*OXA‐23 genes were identified in 45.8% and 41.6%, respectively, of the *K. pneumoniae* isolates analyzed [51].

It is believed that many beta‐lactamases currently found within mobile genetic elements originated from the chromosomes of other bacteria. As occurred with the SHV type variants, which derived from the *K. pneumoniae* chromosomal SHV‐1, and CTX‐M‐type variants, which appear to have originated from the chromosomal CTX‐M of *Kluyvera* spp. [25] This shift in gene presentation, from chromosomal to mobile elements, may explain the emergence of genes like chromosomal *bla*OXA, typically found in *A. baumannii* strains on plasmids widespread in other species.

The presence of isolates harboring multiple genes encoding beta‐lactamases, ranging from 3 to 12 of the genes analyzed, was observed. Studies indicate a high prevalence among *Enterobacteriaceae* of accumulating genes from the *bla* family, primarily those encoding ESBLs, OXA beta‐lactamases, and carbapenemases such as *bla*KPC and *bla*NDM. This phenomenon is more pronounced in hospital environments where selective pressure favors microorganisms carrying resistance genes and where a high rate of gene sharing occurs [52, 53].

This study has some limitations, such as its exclusive focus on beta‐lactam resistance, conferred by genes belonging to the *bla* family, which limits a broader understanding of AMR mechanisms, since many other genes related to a high diverse variety of resistance mechanisms are involved in MDR development [54]. However, it presents a comprehensive methodology, providing a suite of molecular assays to explore resistance to beta‐lactams, one of the largest, most important, and most affected antimicrobial classes, especially when it comes to gram‐negative microorganisms such as *K. pneumoniae*.

## 5. Conclusion

The assays developed met the quality criteria for qPCR reactions using the SYBR Green master mix and were efficient in detecting the beta‐lactam resistance genes from the *bla* family evaluated in this study. The *bla* gene genotypes obtained are relevant and concerning for the local hospital scenario and are compatible with the identified phenotypic profile.

The results of this work demonstrate the high prevalence of AMR in *K. pneumoniae* isolates within the local hospital environment. This phenotypic pattern is of great concern as it limits available therapeutic options, directly impacting patient prognosis. The findings obtained in this work can be used to guide local clinicians, coordinators, and the hospital infection control committee in adopting measures to contain and prevent the spread of resistance genes.

However, further data acquisition is needed to better correlate genotype and phenotype profiles, as well as to include other genes responsible for resistance to additional antibiotic classes. This would be an important improvement before the use of molecular tests, such as those described, for clinical diagnosis. Nonetheless, this work represents a step forward in a scenario where resistance identification will become faster and more efficient.

## Ethics Statement

The ethics approval was approved by the Brazilian National Research Ethics Committee (n° 03300218.2.3001.5045) statement (n° 3.276.113).

## Disclosure

A preprint of the current manuscript has previously been published.

## Conflicts of Interest

The authors declare no conflicts of interest.

## Author Contributions

Lavouisier F.B. Nogueira, Marília S Maia, and Marco A.F. Clementino contributed equally to this work.

## Funding

This study was supported by Conselho Nacional de Desenvolvimento Científico e Tecnológico (10.13039/501100003593, No. 402607/2018 408549/2022‐0) and Fundação Cearense de Apoio ao Desenvolvimento Científico e Tecnológico (10.13039/501100005283, 102/2021 – DINOV).

## Supporting information


**Supporting Information 1** Additional supporting information can be found online in the Supporting Information section. Text S1: Identifiable sequences for each gene included in the study available on the CARD platform. Text S2: Consensus sequences obtained for the gene groups analyzed. Figure S1: Primer′s specificity evaluation through melting curve: (a) SHV, (b) KPC, (c) NDM, (d) TEM, (e) GES, (f) OXA‐23*like*, (g) OXA‐24/40*like*, (h) OXA‐48*like*, (i) OXA‐51*like*, (j) CTX‐M 1.1*like*, (k) CTX‐M 1.2*like*, (l) CTX‐M 2*like*, (m) CTX‐M 8*like*, and (n) CTX‐M 9*like*.

## Data Availability

The authors confirm that all supporting data and protocols have been provided within the article or through supporting data files.
