# Supplementary material for: Insights of β‐Lactams Resistance in Klebsiella pneumoniae Clinical Isolates: A Focus on Molecular Identification of Drug Resistance
Source: Int J Microbiol. 2026 Jan 29;2026:2707907. doi: 10.1155/ijm/2707907 (PMC12853143; doi:10.1155/ijm/2707907)
Supplement: Supplementary file 1 — Supporting Information 1 Additional supporting information can be found online in the Supporting Information section. Text S1: Identifiable sequences for each gene included in the study available on the CARD platform. Text S2: Consensus sequences obtained for the gene groups analyzed. Figure S1: Primer′s specificity evaluation through melting curve: (a) SHV, (b) KPC, (c) NDM, (d) TEM, (e) GES, (f) OXA‐23like, (g) OXA‐24/40like, (h) OXA‐48like, (i) OXA‐51like, (j) CTX‐M 1.1like, (k) CTX‐M 1.2like, (l) CTX‐M 2like, (m) CTX‐M 8like, and (n) CTX‐M 9like. [file IJM-2026-2707907-s001.docx]

**Molecular Insights of β-Lactams Resistance *Klebsiella pneumoniae* Clinical Isolates: A Focus on Multidrug Resistance and Virulence**

Lavouisier F.B. Nogueira^# 1,2^, Marília S. Maia^# 1,2^, Marco A.F. Clementino^# 1,2^, Ila F.N. Lima^3^, Jorge L.N. Rodrigues^3^, Luciana V.C. Fragoso^3^, Glairta S. Costa^3^, Jose Q.S. Filho^1^, Alexandre Havt^1,2^, Deiziane V.S. Costa^5*^, Lyvia M.V.C. Magalhães^1^, Dilza Silva^6^, Nicholas E. Sherman^6^, José K. Sousa^4^ and Aldo A.M. Lima^1,2*^

^1^Institute of Biomedicine, Faculty of Medicine, Federal University of Ceará, Fortaleza-Ceará 60430-270, Brazil.

^2^Postgraduate Medical Microbiology Program, Department of Medicine, Federal University of Ceara, Fortaleza-Ceara 60430-270, Brazil.

^3^Hospital Universitário Walter Cantídio, Federal University of Ceara, Fortaleza-Ceara 60430-270, Brazil.

^4^Department of Gastroenterology, Hepatology and Nutrition, Cincinnati Children’s Hospital Medical Center, Cincinnati, Ohio 45229, United States

^5^Division of Infectious Diseases and International Health, University of Virginia, Charlottesvillle, VA 22908.

^6^Biomolecular Analysis Facility, School of Medicine, University of Virginia, Charlottesvillle, VA 22908.

^#^These authors contributed equally to this work.

## Correspondence: Aldo A.M. Lima ([alima@ufc.br](mailto:alima@ufc.br)); Deiziane V.S. Costa ([dv2t@virginia.edu](mailto:dv2t@virginia.edu)).

**Text S1:** Identifiable sequences for each gene included in the study are available on the CARD platform.

***bla*SHV Gene:**

>gb|FJ668814|77-937|SHV-1 [Klebsiella pneumoniae]; >gb|AF148851|6-866|SHV-2 [Escherichia coli]; >gb|X98102|74-934|SHV-2A [Klebsiella pneumoniae]; >gb|KX092356.1|193-1053|SHV-3 [Klebsiella pneumoniae]; >gb|LT985229.1|36108-36968|SHV-4 [Escherichia coli]; >gb|X55640|112-972|SHV-5 [Klebsiella pneumoniae]; >gb|Y11069|1-780|SHV-6 [Klebsiella pneumoniae]; >gb|U20270|125-985|SHV-7 [Escherichia coli]; >gb|U92041|1-861|SHV-8 [Escherichia coli]; >gb|S82452|121-978|SHV-9 [Klebsiella pneumoniae]; >gb|X98101|74-934|SHV-11 [Klebsiella pneumoniae]; >gb|AJ920369|24-860|SHV-12 [Escherichia coli]; >gb|AF164577|1-861|SHV-13 [Klebsiella pneumoniae]; >gb|AF226622|55-915|SHV-14 [Klebsiella pneumoniae]; >gb|AJ011428|1-861|SHV-15 [Escherichia coli]; >gb|AF072684|118-993|SHV-16 [Klebsiella pneumoniae]; >gb|AF132290|88-948|SHV-18 [Klebsiella pneumoniae]; >gb|AF117743|1-780|SHV-19 [Klebsiella pneumoniae]; >gb|AF117744|1-780|SHV-20 [Klebsiella pneumoniae]; >gb|AF117745|1-780|SHV-21 [Klebsiella pneumoniae]; >gb|AF117746|1-780|SHV-22 [Klebsiella pneumoniae]; >gb|AF117747|1-780|SHV-23 [Klebsiella pneumoniae]; >gb|AB023477|1-861|SHV-24 [Escherichia coli]; >gb|AF208796|1-861|SHV-25 [Klebsiella pneumoniae]; >gb|AF227204|74-934|SHV-26 [Klebsiella pneumoniae]; >gb|AF293345|1-861|SHV-27 [Klebsiella pneumoniae]; >gb|AF299299|1-861|SHV-28 [Klebsiella pneumoniae]; >gb|AF301532|8-868|SHV-29 [Klebsiella pneumoniae]; >gb|AY661885|49-909|SHV-30 [Enterobacter cloacae]; >gb|AY277255|67-927|SHV-31 [Klebsiella pneumoniae]; >gb|AY037778|92-952|SHV-32 [Klebsiella pneumoniae]; >gb|JX268631|1-861|SHV-33 [Klebsiella pneumoniae]; >gb|AY036620|89-949|SHV-34 [Escherichia coli]; >gb|AY070258|1-861|SHV-35 [Klebsiella pneumoniae]; >gb|AF467947|1-861|SHV-36 [Klebsiella pneumoniae]; >gb|AF467948|1-861|SHV-37 [Klebsiella pneumoniae]; >gb|AY079099|149-1009|SHV-38 [Klebsiella pneumoniae]; >gb|AF535128|1-861|SHV-40 [Klebsiella pneumoniae]; >gb|AF535129|1-861|SHV-41 [Klebsiella pneumoniae]; >gb|AF535130|1-861|SHV-42 [Klebsiella pneumoniae]; >gb|AY065991|7-867|SHV-43 [Klebsiella pneumoniae]; >gb|AY259119|117-977|SHV-44 [Klebsiella pneumoniae]; >gb|AF547625|1-861|SHV-45 [Klebsiella pneumoniae]; >gb|AY210887|112-972|SHV-46 [Klebsiella oxytoca]; >gb|AY263404|1-861|SHV-48 [Klebsiella pneumoniae]; >gb|AY528718|414-1274|SHV-49 [Klebsiella pneumoniae]; >gb|AY288915|1-861|SHV-50 [Klebsiella pneumoniae]; >gb|AY289548|1-861|SHV-51 [Klebsiella pneumoniae]; >gb|HQ845196|1-861|SHV-52 [Klebsiella pneumoniae]; >gb|AY590467|1-729|SHV-53 [Klebsiella pneumoniae]; >gb|AJ863560|1-861|SHV-55 [Klebsiella pneumoniae]; >gb|EU586041|1-861|SHV-56 [Klebsiella pneumoniae]; >gb|AY223863|171-1031|SHV-57 [Escherichia coli]; >gb|AY790341|1-861|SHV-59 [Klebsiella pneumoniae]; >gb|AB302939|9-869|SHV-60 [Klebsiella pneumoniae]; >gb|AJ866284|1-861|SHV-61 [Klebsiella pneumoniae]; >gb|AJ866285|1-861|SHV-62 [Klebsiella pneumoniae]; >gb|EU342351|173-1033|SHV-63 [Klebsiella pneumoniae]; >gb|DQ174304|5-865|SHV-64 [Klebsiella pneumoniae]; >gb|DQ174305|5-865|SHV-65 [Klebsiella pneumoniae]; >gb|DQ174306|5-865|SHV-66 [Klebsiella pneumoniae]; >gb|DQ174307|5-865|SHV-67 [Klebsiella pneumoniae]; >gb|DQ174308|5-865|SHV-69 [Klebsiella pneumoniae]; >gb|DQ013287|1-861|SHV-70 [Enterobacter cloacae]; >gb|AM176546|1-861|SHV-71 [Klebsiella pneumoniae]; >gb|AM176547|31-891|SHV-72 [Klebsiella pneumoniae]; >gb|AM176548|17-874|SHV-73 [Klebsiella pneumoniae]; >gb|AM176549|31-891|SHV-74 [Klebsiella pneumoniae]; >gb|AM176550|31-891|SHV-75 [Klebsiella pneumoniae]; >gb|AM176551|31-891|SHV-76 [Klebsiella pneumoniae]; >gb|AM176552|31-891|SHV-77 [Klebsiella pneumoniae]; >gb|AM176553|31-891|SHV-78 [Klebsiella pneumoniae]; >gb|AM176554|31-891|SHV-79 [Klebsiella pneumoniae]; >gb|AM176555|1-861|SHV-80 [Klebsiella pneumoniae]; >gb|AM176556|26-886|SHV-81 [Klebsiella pneumoniae]; >gb|AM176557|1-861|SHV-82 [Klebsiella pneumoniae]; >gb|AM176558|1-861|SHV-83 [Klebsiella pneumoniae]; >gb|AM087453|26-856|SHV-84 [Escherichia coli]; >gb|DQ322460|16-876|SHV-85 [Klebsiella pneumoniae]; >gb|DQ328802|1-861|SHV-86 [Klebsiella pneumoniae]; >gb|DQ193536|1-861|SHV-89 [Klebsiella pneumoniae]; >gb|DQ836922|1-861|SHV-92 [Klebsiella pneumoniae]; >gb|EF373969|1-861|SHV-93 [Klebsiella pneumoniae]; >gb|EF373970|1-861|SHV-94 [Klebsiella pneumoniae]; >gb|EF373972|1-861|SHV-95 [Citrobacter freundii]; >gb|EF373971|1-861|SHV-96 [Acinetobacter baumannii]; >gb|EF373973|1-861|SHV-97 [Enterococcus faecalis]; >gb|EU155018|1-861|SHV-101 [Klebsiella pneumoniae]; >gb|EU024485|1-861|SHV-102 [Escherichia coli]; >gb|EU032604|1-861|SHV-103 [Klebsiella pneumoniae]; >gb|EU274581|1-861|SHV-104 [Klebsiella pneumoniae]; >gb|FJ194944|47-907|SHV-105 [Klebsiella pneumoniae]; >gb|AM941847|1-861|SHV-106 [Klebsiella pneumoniae]; >gb|AM941848|1-861|SHV-107 [Klebsiella pneumoniae]; >gb|HM751100|1-861|SHV-108 [Klebsiella pneumoniae]; >gb|EU418913|17-877|SHV-109 [Klebsiella pneumoniae]; >gb|HQ877615|1-861|SHV-110 [Klebsiella pneumoniae]; >gb|AB372881|9-869|SHV-111 [Klebsiella pneumoniae]; >gb|JF812965|1-861|SHV-120 [Escherichia coli]; >gb|HQ661362|72-932|SHV-121 [Klebsiella pneumoniae]; >gb|HM751103.1|1-864|SHV-122 [Klebsiella pneumoniae]; >gb|GQ390805|1-813|SHV-123 [Klebsiella pneumoniae]; >gb|GQ390806|1-813|SHV-124 [Klebsiella pneumoniae]; >gb|GQ390807|1-813|SHV-125 [Klebsiella pneumoniae]; >gb|GQ390808|1-813|SHV-126 [Escherichia coli]; >gb|GQ390809|1-813|SHV-127 [Klebsiella pneumoniae]; >gb|GU932590|1-861|SHV-128 [Enterobacter cloacae]; >gb|GU827715|1-861|SHV-129 [Escherichia coli]; >gb|AB551737|15-875|SHV-133 [Klebsiella pneumoniae]; >gb|HM559945|1-861|SHV-134 [Klebsiella pneumoniae]; >gb|HQ637576|1-861|SHV-135 [Escherichia coli]; >gb|HQ661363|72-932|SHV-137 [Klebsiella pneumoniae]; >gb|JN051143|1-861|SHV-140 [Klebsiella pneumoniae]; >gb|JQ388884|1-861|SHV-141 [Klebsiella pneumoniae]; >gb|JQ029959|29-889|SHV-142 [Klebsiella pneumoniae]; >gb|JX013655|1-861|SHV-145 [Klebsiella pneumoniae]; >gb|JX121114|1-858|SHV-147 [Klebsiella pneumoniae]; >gb|JX121115|1-858|SHV-148 [Klebsiella pneumoniae]; >gb|JX121116|1-858|SHV-149 [Klebsiella pneumoniae]; >gb|JX121117|1-858|SHV-150 [Klebsiella pneumoniae]; >gb|JX121118|1-858|SHV-151 [Klebsiella pneumoniae]; >gb|JX121119|1-858|SHV-152 [Klebsiella pneumoniae]; >gb|JX121120|1-858|SHV-153 [Klebsiella pneumoniae]; >gb|JX121121|1-858|SHV-154 [Klebsiella pneumoniae]; >gb|JX121122|1-858|SHV-155 [Klebsiella pneumoniae]; >gb|JX121123|1-861|SHV-156 [Klebsiella pneumoniae]; >gb|JX121124|1-861|SHV-157 [Klebsiella pneumoniae]; >gb|JX121125|1-858|SHV-158 [Klebsiella pneumoniae]; >gb|JX121126|1-858|SHV-159 [Klebsiella pneumoniae]; >gb|JX121127|1-861|SHV-160 [Klebsiella pneumoniae]; >gb|JX121128|1-861|SHV-161 [Klebsiella pneumoniae]; >gb|JX121129|1-858|SHV-162 [Klebsiella pneumoniae]; >gb|JX121130|1-858|SHV-163 [Klebsiella pneumoniae]; >gb|JX121131|1-858|SHV-165 [Klebsiella pneumoniae]; >gb|AB733453|4-849|SHV-167 [Klebsiella pneumoniae]; >gb|AM941844|1-861|SHV-98 [Klebsiella pneumoniae]; >gb|AM941845|1-861|SHV-99 [Klebsiella pneumoniae]; >gb|AM941846|1-900|SHV-100 [Klebsiella pneumoniae]; >gb|KJ776406|17-877|SHV-119 [Klebsiella pneumoniae]; >gb|JQ341060.1|882-1742|SHV-143 [Klebsiella pneumoniae]; >gb|JQ926986|1-861|SHV-144 [Klebsiella pneumoniae]; >gb|HE981194|1-861|SHV-164 [Klebsiella pneumoniae]; >gb|KP050487.1|1-861|SHV-180 [Klebsiella pneumoniae]; >gb|HG934764|1-864|SHV-183 [Enterobacter cloacae]; >gb|JX870080|1-861|SHV-168 [Klebsiella pneumoniae]; >gb|KF513177|1-861|SHV-172 [Klebsiella pneumoniae]; >gb|KF513178|1-861|SHV-173 [Klebsiella pneumoniae]; >gb|KF705209|1-861|SHV-178 [Klebsiella pneumoniae]; >gb|KF705208|1-861|SHV-179 [Klebsiella pneumoniae]; >gb|KP050489|1-861|SHV-182 [Klebsiella pneumoniae]; >gb|KM233164|1-861|SHV-185 [Klebsiella pneumoniae]; >gb|KM233165|1-861|SHV-186 [Klebsiella pneumoniae]; >gb|LN515533|1-867|SHV-187 [Klebsiella pneumoniae]; >gb|LN515534|1-873|SHV-188 [Klebsiella pneumoniae]; >gb|KP050494|1-861|SHV-189 [Klebsiella pneumoniae].

***bla*TEM Gene:**

>gb|AL513383|161911-162771|TEM-1 [Salmonella enterica subsp. enterica serovar Typhi str. CT18]; >gb|X54606|215-1075|TEM-2 [Pseudomonas aeruginosa]; >gb|X64523.1|477-1337|TEM-3 [Klebsiella pneumoniae]; >gb|LK391770.1|19708-20568|TEM-4 [Klebsiella pneumoniae]; >gb|X57972|340-1200|TEM-6 [Escherichia coli]; >gb|AF527798.1|1-785|TEM-7 [Escherichia coli]; >gb|X65252|176-1036|TEM-8 [Klebsiella pneumoniae]; >gb|AF093512|198-1058|TEM-10 [Morganella morganii]; >gb|AY874537|178-1038|TEM-11 [Proteus mirabilis]; >gb|M88143|368-1228|TEM-12 [Klebsiella oxytoca]; >gb|AM849805|263-1123|TEM-15 [Haemophilus parainfluenzae]; >gb|X65254|176-1036|TEM-16 [Klebsiella pneumoniae]; >gb|Y14574|1-861|TEM-17 [Capnocytophaga ochracea]; >gb|JX042489|142-1002|TEM-19 [Acinetobacter baumannii]; >gb|Y17581|79-936|TEM-20 [Klebsiella pneumoniae]; >gb|Y17582|1-858|TEM-21 [Klebsiella pneumoniae]; >gb|Y17583|214-1071|TEM-22 [Klebsiella pneumoniae]; >gb|X65253|176-1036|TEM-24 [Klebsiella pneumoniae]; >gb|NG_050256.1|101-961|TEM-26 [Enterobacteriaceae]; >gb|U37195|76-936|TEM-28 [Escherichia coli]; >gb|Y17584|1-858|TEM-29 [Escherichia coli]; >gb|AJ437107|209-1069|TEM-30 [Escherichia coli]; >gb|GU371926|50305-51165|TEM-33 [Escherichia coli]; >gb|KC292503|4335-5195|TEM-34 [Haemophilus parainfluenzae]; >gb|FR717535|1-861|TEM-40 [Escherichia coli]; >gb|X98047|1-844|TEM-42 [Pseudomonas aeruginosa]; >gb|U95363|1-861|TEM-43 [Klebsiella pneumoniae]; >gb|X95401|209-1069|TEM-45 [Escherichia coli]; >gb|Y10279|1-861|TEM-47 [Klebsiella pneumoniae]; >gb|Y10280|1-861|TEM-48 [Klebsiella pneumoniae]; >gb|Y10281|1-861|TEM-49 [Escherichia coli]; >gb|Y13612|1-861|TEM-52 [Klebsiella pneumoniae]; >gb|AF104441|194-1054|TEM-53 [Klebsiella pneumoniae]; >gb|AF104442|194-1054|TEM-54 [Escherichia coli]; >gb|DQ286729|1-861|TEM-55 [Escherichia coli]; >gb|FJ405211|1-861|TEM-57 [Escherichia coli]; >gb|AF062386|31-862|TEM-59 [Klebsiella oxytoca]; >gb|AF047171|136-996|TEM-60 [Providencia stuartii]; >gb|AF332513|104-964|TEM-63 [Escherichia coli]; >gb|AF091113|451-1311|TEM-67 [Proteus mirabilis]; >gb|AJ239002|1-861|TEM-68 [Klebsiella pneumoniae]; >gb|AF188199|215-1075|TEM-70 [Escherichia coli]; >gb|AF203816|211-1071|TEM-71 [Klebsiella pneumoniae]; >gb|AF157553|148-1008|TEM-72 [Morganella morganii]; >gb|AJ012256|209-1069|TEM-73 [Proteus mirabilis]; >gb|AY130284|1-785|TEM-75 [Klebsiella pneumoniae]; >gb|AF190694|209-1069|TEM-76 [Escherichia coli]; >gb|AF190693|209-1069|TEM-78 [Escherichia coli]; >gb|AF190692|209-1069|TEM-79 [Escherichia coli]; >gb|AF347054|209-1069|TEM-80 [Enterobacter cloacae]; >gb|AF427127|209-1069|TEM-81 [Escherichia coli]; >gb|AF427128|209-1069|TEM-82 [Escherichia coli]; >gb|AF427129|209-1069|TEM-83 [Escherichia coli]; >gb|AF427130|209-1069|TEM-84 [Escherichia coli]; >gb|AJ277414|1-861|TEM-85 [Klebsiella pneumoniae]; >gb|AJ277415|1-861|TEM-86 [Klebsiella pneumoniae]; >gb|AF250872|1-861|TEM-87 [Proteus mirabilis]; >gb|AY027590|113-973|TEM-88 [Klebsiella pneumoniae]; >gb|AY039040|189-1022|TEM-89 [Proteus mirabilis]; >gb|AF351241|90-950|TEM-90 [Escherichia coli]; >gb|AB049569|1-861|TEM-91 [Escherichia coli]; >gb|AF143804|1-861|TEM-92 [Proteus mirabilis]; >gb|AJ318093|1-861|TEM-93 [Escherichia coli]; >gb|AJ318094|1-861|TEM-94 [Escherichia coli]; >gb|AJ308558|182-1042|TEM-95 [Escherichia coli]; >gb|AY092401|1-861|TEM-96 [Escherichia coli]; >gb|AF495873|1-861|TEM-101 [Escherichia coli]; >gb|AY040093|69-929|TEM-102 [Plasmid pWW100]; >gb|AF516719|215-1075|TEM-104 [Klebsiella pneumoniae]; >gb|AF516720|215-1075|TEM-105 [Escherichia coli]; >gb|AY101578|215-1075|TEM-106 [Escherichia coli]; >gb|AY101764|207-1067|TEM-107 [Klebsiella pneumoniae]; >gb|AF506748|39-899|TEM-108 [Salmonella enterica subsp. enterica serovar Typhimurium]; >gb|AY628175|211-1071|TEM-109 [Escherichia coli]; >gb|AY072920|1-861|TEM-110 [Klebsiella pneumoniae]; >gb|AF468003|1-861|TEM-111 [Escherichia coli]; >gb|AY589493|167-1027|TEM-112 [Escherichia coli]; >gb|AY589494|194-1054|TEM-113 [Proteus mirabilis]; >gb|AY589495|182-1042|TEM-114 [Klebsiella aerogenes]; >gb|AF535127|209-1069|TEM-115 [Klebsiella pneumoniae]; >gb|U36911.1|1430-2290|TEM-116 [Staphylococcus aureus]; >gb|AY130282|1-764|TEM-117 [Escherichia coli]; >gb|AY130285|1-785|TEM-118 [Klebsiella oxytoca]; >gb|AY243512|209-1069|TEM-120 [Klebsiella oxytoca]; >gb|AY271267|1-861|TEM-121 [Escherichia coli]; >gb|AY307100|1-861|TEM-122 [Escherichia coli]; >gb|AY327539|1-858|TEM-123 [Proteus mirabilis]; >gb|AY327540|1-858|TEM-124 [Morganella morganii]; >gb|AY628176|76-936|TEM-125 [Escherichia coli]; >gb|AY628199|204-1064|TEM-126 [Escherichia coli]; >gb|AY368236|1-861|TEM-127 [Escherichia coli]; >gb|AY368237|1-861|TEM-128 [Escherichia coli]; >gb|AJ746225|1-861|TEM-129 [Klebsiella oxytoca]; >gb|AJ866988|1-861|TEM-130 [Klebsiella pneumoniae]; >gb|AY436361|132-992|TEM-131 [Salmonella enterica subsp. enterica serovar Typhimurium]; >gb|AY491682|1-861|TEM-132 [Klebsiella pneumoniae]; >gb|AY528425|1-861|TEM-133 [Klebsiella pneumoniae]; >gb|AY574271|1-861|TEM-134 [Citrobacter koseri]; >gb|AJ634602.1|7840-8700|TEM-135 [Salmonella enterica subsp. enterica serovar Typhimurium]; >gb|AY826417|1-861|TEM-136 [Klebsiella pneumoniae]; >gb|AM286274|1-861|TEM-137 [Shigella sonnei]; >gb|AY853593|215-1075|TEM-138 [Salmonella enterica]; >gb|DQ072853|1-861|TEM-139 [Klebsiella pneumoniae]; >gb|AY956335|39-899|TEM-141 [Enterobacter cloacae]; >gb|DQ388882|1-861|TEM-142 [Escherichia coli]; >gb|DQ075245|218-1078|TEM-143 [Escherichia coli]; >gb|AM049399|1-861|TEM-144 [Salmonella enterica subsp. enterica serovar Derby]; >gb|DQ105528|1-861|TEM-145 [Escherichia coli]; >gb|DQ105529|1-861|TEM-146 [Escherichia coli]; >gb|DQ279850|1-861|TEM-147 [Pseudomonas aeruginosa]; >gb|AM087454|209-1069|TEM-148 [Escherichia coli]; >gb|DQ369751|1-861|TEM-149 [Klebsiella aerogenes]; >gb|AM183304|209-1069|TEM-150 [Escherichia coli]; >gb|DQ834729|206-1066|TEM-151 [Escherichia coli]; >gb|DQ834728|206-1066|TEM-152 [Escherichia coli]; >gb|FJ807656|1-861|TEM-154 [Escherichia coli]; >gb|DQ679961|115-975|TEM-155 [Proteus mirabilis]; gb|AM941159|209-1069|TEM-156 [Proteus mirabilis]; >gb|DQ909059|1-861|TEM-157 [Enterobacter cloacae]; >gb|EF534736|214-1074|TEM-158 [Escherichia coli]; >gb|EF136376|1-861|TEM-159 [Proteus mirabilis]; >gb|EF136377|1-861|TEM-160 [Proteus mirabilis]; >gb|EF468463|67-927|TEM-162 [Acinetobacter haemolyticus]; >gb|EU815939|1-861|TEM-163 [Escherichia coli]; >gb|EU274580|215-1075|TEM-164 [Klebsiella pneumoniae]; >gb|FJ197316|1-861|TEM-166 [Escherichia coli]; >gb|FJ360884|214-1074|TEM-167 [Escherichia coli]; >gb|FJ919776|209-1069|TEM-168 [Escherichia coli]; >gb|FJ873740|1-858|TEM-169 [Salmonella enterica subsp. enterica serovar Infantis]; >gb|GQ149347|5270-6130|TEM-171 [Escherichia coli]; >gb|GU550123|145-1005|TEM-176 [Escherichia coli]; >gb|FN652295|1-861|TEM-177 [Proteus mirabilis]; >gb|X97254|154-1011|TEM-178 [Serratia marcescens]; >gb|NG_050218.1|1-1061|TEM-181 [Bacteria]; >gb|HQ529916|111-971|TEM-183 [Klebsiella pneumoniae]; >gb|JN227084|309-1169|TEM-186 [Escherichia coli]; >gb|HM246246|212-1069|TEM-187 [Proteus mirabilis]; >gb|JN211012|214-1074|TEM-188 [Salmonella enterica]; >gb|JN254627|1-861|TEM-189 [Escherichia coli]; >gb|JN416112|1-861|TEM-190 [Escherichia coli]; >gb|KY432484.1|1-861|TEM-191 [Acinetobacter baumannii]; >gb|JF949915|1-754|TEM-192 [Klebsiella pneumoniae]; >gb|JN935135|1-861|TEM-193 [Acinetobacter baumannii]; >gb|JN935136|1-861|TEM-194 [Acinetobacter baumannii]; >gb|JN935137|1-861|TEM-195 [Acinetobacter baumannii]; >gb|HQ877606|1-861|TEM-197 [Klebsiella pneumoniae]; >gb|AB700703|162-1022|TEM-198 [Klebsiella pneumoniae]; >gb|JX050178|1-853|TEM-199 [Proteus mirabilis]; >gb|KC149518|1-861|TEM-153 [Escherichia coli]; >gb|HQ317449|1-861|TEM-182 [Haemophilus parainfluenzae]; >gb|JF795538|1-861|TEM-185 [Escherichia coli]; >gb|FR848831|1-861|TEM-184 [Escherichia coli]; >gb|JQ034306|1-861|TEM-196 [Shigella sonnei]; >gb|JX310327|1-861|TEM-201 [Escherichia coli]; >gb|KC900516|1-858|TEM-205 [Pseudomonas aeruginosa]; >gb|KC783461|1-861|TEM-206 [Escherichia coli]; >gb|KC818234|1-861|TEM-207 [Escherichia coli]; >gb|KC865667|184-1044|TEM-208 [Escherichia coli]; >gb|KF240808|1-861|TEM-209 [Klebsiella pneumoniae]; >gb|KF513179|1-861|TEM-211 [Proteus mirabilis]; >gb|KF663615|1-858|TEM-213 [Pseudomonas aeruginosa]; >gb|KP050491|1-861|TEM-214 [Escherichia coli]; >gb|KP050492|1-861|TEM-215 [Escherichia coli]; >gb|KF944358|1-861|TEM-216 [Escherichia coli]; >gb|HG934763|1-861|TEM-217 [Enterobacter cloacae]; >gb|KM114268|1-861|TEM-219 [Escherichia coli]; >gb|KM998962.1|1-861|TEM-220 [Neisseria gonorrhoeae].

***bla*NDM Gene:**

>gb|FN396876|2407-3219|NDM-1 [Klebsiella pneumoniae]; >gb|JF703135|1-813|NDM-2 [Acinetobacter baumannii]; >gb|JN104597|115-927|NDM-5 [Escherichia coli]; >gb|JQ734687|1-813|NDM-3 [Escherichia coli]; >gb|JQ348841|1-813|NDM-4 [Escherichia coli]; >gb|JN967644|1-813|NDM-6 [Escherichia coli]; >gb|JX262694|1-813|NDM-7 [Escherichia coli]; >gb|AB744718|1-813|NDM-8 [Escherichia coli]; >gb|KC999080|380-1192|NDM-9 [Klebsiella pneumoniae subsp. pneumoniae]; >gb|KF361506|1-813|NDM-10 [Klebsiella pneumoniae subsp. pneumoniae]; >gb|KP265939.1|1-813|NDM-11 [Escherichia coli]; >gb|AB926431|511-1323|NDM-12 [Escherichia coli]; >gb|LC012596|3586-4398|NDM-13 [Escherichia coli]; >gb|KM210086.1|9068-9880|NDM-14 [Acinetobacter lwoffii]; >gb|KP735848.1|1-813|NDM-15 [Escherichia coli]; >gb|KP862821.1|1-813|NDM-16 [Klebsiella pneumoniae]; >gb|KX812714|1-813|NDM-17 [Escherichia coli]; >gb|KY503030.1|1-828|NDM-18 [Escherichia coli]; >gb|MF370080.1|1-813|NDM-19 [Escherichia coli]; >gb|KY654092.1|1-813|NDM-20 [Escherichia coli]; >gb|MG183694.1|1-813|NDM-21 [Escherichia coli]; >gb|MH243357.1|1-813|NDM-22 [Enterobacter cloacae]; >gb|MH450214.1|1-813|NDM-23 [Klebsiella pneumoniae]; >gb|MH450215.1|1-813|NDM-24 [Providencia stuartii]; >gb|MH986670.1|1-813|NDM-25 [Klebsiella pneumoniae]; >gb|MK105832.1|1-813|NDM-27 [Escherichia coli]; >gb|MK425035.1|1-813|NDM-28 [Klebsiella pneumoniae].

***bla*KPC Gene:**

blaKPC-10,"NG_049243.1"; blaKPC-100,"NG_081070.1"; blaKPC-101,"NG_088394.1"; blaKPC-102,"NG_078063.1"; blaKPC-103,"NG_078051.1"; blaKPC-104,"NG_078052.1"; blaKPC-105,"NG_078054.1"; blaKPC-106,"NG_078056.1"; blaKPC-107,"NG_078053.1"; blaKPC-108,"NG_078055.1"; blaKPC-109,"NG_149659.1"; blaKPC-11,"NG_049244.1"; blaKPC-110,"NG_088395.1"; blaKPC-111,"NG_081791.1"; blaKPC-112,"NG_079230.1"; blaKPC-113,"NG_079888.1"; blaKPC-114,"NG_079889.1"; blaKPC-115,"NG_079890.1"; blaKPC-116,"NG_079891.1"; blaKPC-117,"NG_079892.1"; blaKPC-118,"NG_079893.1"; blaKPC-119,"NG_079894.1"; blaKPC-12,"NG_049245.1"; blaKPC-120,"NG_079895.1"; blaKPC-121,"NG_079896.1"; blaKPC-122,"NG_079897.1"; blaKPC-123,"NG_079898.1"; blaKPC-124,"NG_203393.1"; blaKPC-125,"NG_080778.1"; blaKPC-126,"NG_080779.1"; blaKPC-127,"NG_081071.1"; blaKPC-128,"NG_081072.1"; blaKPC-129,"NG_203394.1"; blaKPC-13,"NG_049246.1"; blaKPC-130,"NG_081699.1"; blaKPC-131,"NG_081700.1"; blaKPC-132,"NG_081783.1"; blaKPC-133,"NG_081784.1"; blaKPC-134,"NG_088396.1"; blaKPC-135,"NG_088397.1"; blaKPC-136,"NG_157007.1"; blaKPC-137,"NG_242182.1"; blaKPC-138,"NG_088398.1"; blaKPC-139,"NG_088399.1"; blaKPC-14,"NG_049247.1"; blaKPC-140,"NG_088400.1"; blaKPC-141,"NG_088401.1"; blaKPC-142,"NG_088402.1"; blaKPC-143,"NG_088403.1"; blaKPC-144,"NG_088404.1"; blaKPC-145,"NG_148622.1"; blaKPC-146,"NG_148623.1"; blaKPC-147,"NG_148624.1"; blaKPC-148,"NG_148625.1"; blaKPC-149,"NG_242293.1"; blaKPC-15,"NG_049248.1"; blaKPC-150,"NG_242294.1"; blaKPC-151,"NG_148626.1"; blaKPC-152,"NG_242295.1"; blaKPC-153,"NG_148627.1"; blaKPC-154,"NG_231545.1"; blaKPC-155,"NG_149660.1"; blaKPC-156,"NG_149661.1"; blaKPC-157,"NG_149662.1"; blaKPC-158,"NG_228670.1"; blaKPC-159,"NG_157008.1"; blaKPC-16,"NG_049249.1"; blaKPC-160,"NG_157009.1"; blaKPC-161,"NG_157010.1"; blaKPC-162,"NG_157011.1"; blaKPC-163,"NG_157012.1"; blaKPC-164,"NG_157013.1"; blaKPC-165,"NG_157014.1"; blaKPC-166,"NG_157015.1"; blaKPC-167,"NG_157016.1"; blaKPC-168,"NG_242183.1"; blaKPC-169,"NG_242550.1"; blaKPC-17,"NG_049250.1"; blaKPC-170,"NG_231546.1"; blaKPC-171,"NG_242551.1"; blaKPC-172,"NG_242552.1"; blaKPC-173,"NG_242553.1"; blaKPC-174,"NG_242554.1"; blaKPC-175,"NG_242555.1"; blaKPC-176,"NG_242184.1"; blaKPC-177,"NG_242556.1"; blaKPC-178,"NG_203395.1"; blaKPC-179,"NG_203396.1"; blaKPC-18,"NG_049251.1"; blaKPC-180,"NG_203397.1"; blaKPC-181,"NG_228671.1"; blaKPC-182,"NG_228672.1"; blaKPC-183,"NG_228673.1"; blaKPC-184,"NG_228674.1"; blaKPC-185,"NG_231547.1"; blaKPC-186,"NG_231548.1"; blaKPC-187,"NG_231549.1"; blaKPC-189,"NG_231550.1"; blaKPC-19,"NG_049252.1"; blaKPC-190,"NG_231551.1"; blaKPC-191,"NG_231552.1"; blaKPC-192,"NG_231553.1"; blaKPC-193,"NG_242185.1"; blaKPC-194,"NG_242186.1"; blaKPC-195,"NG_242187.1"; blaKPC-196,"NG_242188.1"; blaKPC-197,"NG_242189.1"; blaKPC-2,"NG_049253.1"; blaKPC-201,"NG_242190.1"; blaKPC-202,"NG_242296.1"; blaKPC-203,"NG_242297.1"; blaKPC-204,"NG_242298.1"; blaKPC-205,"NG_242299.1"; blaKPC-206,"NG_242300.1"; blaKPC-207,"NG_242301.1"; blaKPC-208,"NG_242302.1"; blaKPC-209,"NG_242557.1"; blaKPC-21,"NG_049254.1"; blaKPC-211,"NG_242558.1"; blaKPC-212,"NG_242559.1"; blaKPC-213,"NG_242560.1"; blaKPC-214,"NG_242561.1"; blaKPC-215,"NG_242562.1"; blaKPC-216,"NG_242563.1"; blaKPC-22,"NG_049255.1"; blaKPC-23,"NG_060569.1"; blaKPC-24,"NG_049256.1"; blaKPC-25,"NG_051167.1"; blaKPC-26,"NG_051469.1"; blaKPC-27,"NG_052862.1"; blaKPC-28,"NG_052581.1"; blaKPC-29,"NG_055580.1"; blaKPC-3,"NG_049257.1"; blaKPC-30,"NG_054685.1"; blaKPC-31,"NG_055494.1"; blaKPC-32,"NG_055495.1"; blaKPC-33,"NG_056170.1"; blaKPC-34,"NG_057447.1"; blaKPC-35,"NG_060524.1"; blaKPC-36,"NG_061389.1"; blaKPC-37,"NG_061612.1"; blaKPC-38,"NG_062357.1"; blaKPC-39,"NG_063841.1"; blaKPC-4,"NG_049258.1"; blaKPC-40,"NG_064726.1"; blaKPC-41,"NG_065876.1"; blaKPC-42,"NG_064727.1"; blaKPC-43,"NG_064728.1"; blaKPC-44,"NG_065427.1"; blaKPC-45,"NG_065877.1"; blaKPC-46,"NG_065878.1"; blaKPC-47,"NG_074714.1"; blaKPC-48,"NG_074715.1"; blaKPC-49,"NG_071203.1"; blaKPC-5,"NG_049259.1"; blaKPC-50,"NG_068507.1"; blaKPC-51,"NG_067224.1”; blaKPC-52,"NG_067225.1"; blaKPC-53,"NG_068176.1"; blaKPC-54,"NG_067226.1"; blaKPC-55,"NG_068177.1"; blaKPC-56,"NG_068016.1"; blaKPC-57,"NG_068508.1"; blaKPC-58,"NG_070177.1"; blaKPC-59,"NG_070178.1"; blaKPC-6,"NG_049260.1"; blaKPC-60,"NG_070179.1"; blaKPC-61,"NG_070180.1"; blaKPC-62,"NG_073465.1"; blaKPC-63,"NG_073466.1"; blaKPC-64,"NG_073467.1"; blaKPC-65,"NG_073468.1"; blaKPC-66,"NG_070739.1"; blaKPC-67,"NG_074716.1"; blaKPC-68,"NG_074717.1"; blaKPC-69,"NG_074718.1"; blaKPC-7,"NG_049261.1"; blaKPC-70,"NG_074719.1"; blaKPC-71,"NG_070895.1"; blaKPC-72,"NG_070740.1"; blaKPC-73,"NG_070741.1"; blaKPC-74,"NG_070742.1"; blaKPC-75,"NG_070743.1"; blaKPC-76,"NG_070896.1"; blaKPC-77,"NG_070897.1"; blaKPC-78,"NG_071204.1"; blaKPC-79,"NG_071205.1"; blaKPC-8,"NG_049262.1"; blaKPC-80,"NG_073469.1"; blaKPC-81,"NG_073470.1"; blaKPC-82,"NG_073471.1"; blaKPC-83,"NG_079231.1"; blaKPC-84,"NG_074720.1"; blaKPC-85,"NG_074721.1"; blaKPC-86,"NG_074722.1"; blaKPC-87,"NG_074723.1"; blaKPC-88,"NG_074724.1"; blaKPC-89,"NG_079232.1"; blaKPC-90,"NG_076666.1"; blaKPC-91,"NG_076667.1"; blaKPC-92,"NG_079233.1"; blaKPC-93,"NG_080780.1"; blaKPC-94,"NG_076680.1"; blaKPC-95,"NG_076681.1"; blaKPC-96,"NG_078037.1"; blaKPC-97,"NG_078038.1"; blaKPC-98,"NG_078032.1"; blaKPC-99,"NG_088405.1"

***bla*GES Gene:**

>gb|AF156486|1332-2195|GES-1 [Klebsiella pneumoniae]; >gb|AF326355|1-864|GES-2 [Pseudomonas aeruginosa]; >gb|AB113580|1330-2193|GES-3 [Klebsiella pneumoniae]; >gb|AB116260|1330-2193|GES-4 [Klebsiella pneumoniae]; >gb|AY494717|1-864|GES-5 [Escherichia coli]; >gb|AY494718|1-864|GES-6 [Klebsiella pneumoniae]; >gb|AY260546|4478-5341|GES-7 [Escherichia coli]; >gb|AF329699|373-1236|GES-8 [Pseudomonas aeruginosa]; >gb|AY920928|2690-3553|GES-9 [Pseudomonas aeruginosa]; >gb|FJ820124|1124-1987|GES-10 [uncultured bacterium]; >gb|FJ854362|702-1565|GES-11 [Acinetobacter baumannii]; >gb|FN554543|1-864|GES-12 [Acinetobacter baumannii]; >gb|GU169702|609-1472|GES-13 [Pseudomonas aeruginosa]; >gb|GU207844|1-864|GES-14 [Acinetobacter baumannii]; >gb|GU208678|1-864|GES-15 [Pseudomonas aeruginosa]; >gb|HM173356|512-1375|GES-16 [Serratia marcescens]; >gb|HQ874631|1-864|GES-17 [Escherichia coli]; >gb|JQ028729|1-864|GES-18 [Pseudomonas aeruginosa]; >gb|JN596280|1919-2782|GES-19 [Escherichia coli]; >gb|JN596280|2848-3711|GES-20 [Escherichia coli]; >gb|JQ772478|141-1004|GES-21 [uncultured bacterium]; >gb|JX023441|38-901|GES-22 [Acinetobacter baumannii]; >gb|KF179354|1-861|GES-23 [Pseudomonas aeruginosa]; >gb|AB901141|1-864|GES-24 [Acinetobacter baumannii]; >gb|KP096411|1-864|GES-26 [Pseudomonas aeruginosa].

***bla*CTX-M Gene:**

CTX-M 1.1*like* Subgroup:

>gb|Y10278|1-876|CTX-M-3 [Citrobacter freundii]; >gb|AY044436|1436-2311|CTX-M-15 [Escherichia coli]; >gb|AY080894|4-879|CTX-M-22 [Klebsiella pneumoniae]; >gb|AY238472.1|13-888|CTX-M-33 [Escherichia coli]; >gb|DQ061159|347-1222|CTX-M-42 [Escherichia coli]; >gb|DQ303459|2175-3050|CTX-M-54 [Klebsiella pneumoniae]; >gb|DQ885477|1-876|CTX-M-55 [Escherichia coli]; >gb|EF576988|240-1115|CTX-M-66 [Proteus mirabilis]; >gb|FJ815436|196-1071|CTX-M-71 [Klebsiella pneumoniae]; >gb|AY847148|9-884|CTX-M-72 [Klebsiella pneumoniae]; >gb|DQ256091|99-974|CTX-M-82 [Escherichia coli]; >gb|NZ_SQNR01000043.1|1506-2381|CTX-M-88 [Enterobacteriaceae]; >gb|HQ398214|250-1125|CTX-M-101 [Escherichia coli]; >gb|HG423149|40-915|CTX-M-103 [Escherichia coli]; >gb|GQ351346|1-876|CTX-M-114 [Providencia rettgeri]; >gb|JN227085|352-1227|CTX-M-117 [Escherichia coli]; >gb|KC107824|1-876|CTX-M-139 [Escherichia coli]; >gb|KF240809|1-876|CTX-M-142 [Escherichia coli]; >gb|KJ020573|275-1150|CTX-M-144 [Escherichia coli]; >gb|AJ704396|1-876|CTX-M-96 [Klebsiella pneumoniae]; >gb|EF219134|2126-3001|CTX-M-62 [Klebsiella pneumoniae]; >gb|KM211509|1-876|CTX-M-156 [Klebsiella pneumoniae]; >gb|KM211510|1-876|CTX-M-157 [Klebsiella pneumoniae]; >gb|KP681697.1|1-876|CTX-M-162 [Klebsiella oxytoca]; >gb|KP681698.1|1-876|CTX-M-163 [Escherichia coli]; >gb|KP727571.1|1-876|CTX-M-164 [Proteus mirabilis]; >gb|DQ223685|1-876|CTX-M-52 [Klebsiella pneumoniae]; >gb|EU202673|1-876|CTX-M-80 [Klebsiella pneumoniae]; >gb|KC351754|158-1033|CTX-M-136 [Proteus mirabilis]; >gb|KM211508|1-876|CTX-M-155 [Klebsiella pneumoniae]; >gb|JF966749|158-1033|CTX-M-116 [Proteus mirabilis]; >gb|AY005110|1-846|CTX-M-11 [Klebsiella pneumoniae]; >gb|AJ549244|1-876|CTX-M-28 [Escherichia coli]; >gb|JF274244.1|1-864|CTX-M-107 [Shigella sp. SH219]; >gb|JF274245|1-864|CTX-M-108 [Shigella sp. SH223]; >gb|JF274248.1|1-865|CTX-M-109 [Shigella sp. SH361]; >gb|EU402393|1-876|CTX-M-69 [Escherichia coli]; >gb|EF426798|1-876|CTX-M-79 [Escherichia coli]; >gb|AY267213|1-876|CTX-M-29 [Escherichia coli]; >gb|AY292654|1-876|CTX-M-30 [Citrobacter freundii]; >gb|AF305837|1-876|CTX-M-12 [Klebsiella pneumoniae]; >gb|AM411407|1-876|CTX-M-60 [Klebsiella pneumoniae].

CTX-M 1.2*like* Subgroup:

>gb|X92506|1-876|CTX-M-1 [Escherichia coli]; >gb|AJ557142|1-876|CTX-M-32 [Escherichia coli]; >gb|AB177384|1-876|CTX-M-36 [Escherichia coli]; >gb|EF210159|1-876|CTX-M-58 [Escherichia coli]; >gb|EF219142|11-886|CTX-M-61 [Salmonella enterica subsp. enterica serovar Typhimurium]; >gb|KM211691|1-876|CTX-M-158 [Escherichia coli]; >gb|LN830266.1|1-876|CTX-M-166 [Escherichia coli]; >gb|AF488377|63-938|CTX-M-23 [Escherichia coli]; >gb|AY598759|1-876|CTX-M-10 [Klebsiella pneumoniae]; >gb|AY515297|1-876|CTX-M-34 [Escherichia coli]; >gb|DQ268764|5892-6767|CTX-M-53 [Salmonella enterica subsp. enterica serovar Westhampton]; >gb|AY649755|18-893|CTX-M-37 [Enterobacter cloacae]; >gb|EU177100|5-880|CTX-M-68 [Klebsiella sp. ARS06-441]; >gb|JN790864|239-1114|CTX-M-123 [Escherichia coli]; >gb|JX313020|1-876|CTX-M-132 [Escherichia coli]; >gb|AB284167|226-1101|CTX-M-64 [Shigella sonnei].

CTX-M 2*like* Subgroup:

>gb|DQ125241|1-876|CTX-M-2 [Escherichia coli]; >gb|AJ416344|304-1179|CTX-M-20 [Proteus mirabilis]; >gb|AJ567481|1-876|CTX-M-31 [Providencia sp. 4440]; >gb|AB176534|1-876|CTX-M-35 [Klebsiella oxytoca]; >gb|EF374097|1-876|CTX-M-56 [Escherichia coli]; >gb|DQ408762|1-876|CTX-M-59 [Klebsiella pneumoniae]; >gb|GU127598|1-876|CTX-M-92 [Escherichia coli]; >gb|JN969893.3|2531-3406|CTX-M-131 [Providencia rettgeri]; >gb|KC964871|1-876|CTX-M-141 [Klebsiella pneumoniae]; >gb|DQ102702|1-876|CTX-M-43 [Acinetobacter baumannii]; >gb|D37830|91-966|CTX-M-44 [Escherichia coli]; >gb|KP727572.1|1-876|CTX-M-165 [Klebsiella pneumoniae]; >gb|KJ911020|112-987|CTX-M-115 [Acinetobacter baumannii]; >gb|JQ429324|1-876|CTX-M-124 [Escherichia coli]; >gb|U95364|6-881|CTX-M-5 [Salmonella enterica subsp. enterica serovar Typhimurium]; >gb|AM982520|5549-6424|CTX-M-76 [Kluyvera ascorbata]; >gb|AM982521|1912-2787|CTX-M-77 [Kluyvera ascorbata]; >gb|FN813245|1912-2787|CTX-M-95 [Kluyvera ascorbata]; >gb|Y14156|1-876|CTX-M-4 [Salmonella enterica subsp. enterica serovar Typhimurium]; >gb|AJ005045|1-876|CTX-M-7 [Salmonella enterica subsp. enterica serovar Typhimurium]; >gb|AJ005044|1-876|CTX-M-6 [Salmonella enterica subsp. enterica serovar Typhimurium]; >gb|GQ149243|1-873|CTX-M-74 [Enterobacter cloacae]; >gb|GQ149244|1-873|CTX-M-75 [Providencia stuartii].

CTX-M 8*like* Subgroup :

>gb|AY157676|1-876|CTX-M-26 [Klebsiella pneumoniae]; >gb|AY954516|1-876|CTX-M-39 [Escherichia coli]; >gb|HM167760|1-876|CTX-M-94 [Escherichia coli]; >gb|FR682582|1-876|CTX-M-100 [Escherichia coli]; >gb|DQ023162|1-876|CTX-M-41 [Proteus mirabilis]; >gb|AF518567|2321-3196|CTX-M-25 [Escherichia coli]; >gb|FJ971899|31-906|CTX-M-89 [Proteus mirabilis]; >gb|KP050493.1|1-876|CTX-M-160 [Proteus mirabilis]; >gb|GQ870432|31-906|CTX-M-91 [Proteus mirabilis]; >gb|AM982522|1-876|CTX-M-78 [Kluyvera georgiana]; >gb|KJ461948|4-876|CTX-M-152 [Kluyvera sp. MRB7]; >gb|AY750914.2|207-1079|CTX-M-40 [Escherichia coli]; >gb|AB205197|4-876|CTX-M-63 [Klebsiella pneumoniae]; >gb|AF189721|274-1149|CTX-M-8 [Citrobacter amalonaticus].

CTX-M 9*like* Subgroup:

>gb|AF252622|1741-2616|CTX-M-14 [Escherichia coli]; >gb|AF325134|1-876|CTX-M-19 [Klebsiella pneumoniae]; >gb|AY143430|1-876|CTX-M-24 [Klebsiella pneumoniae]; >gb|AY156923|1-876|CTX-M-27 [Escherichia coli]; >gb|AY822595|23-898|CTX-M-38 [Klebsiella pneumoniae]; >gb|AY847143|83-958|CTX-M-47 [Escherichia coli]; >gb|AY847144|82-957|CTX-M-48 [Klebsiella pneumoniae]; >gb|AY847145|82-957|CTX-M-49 [Klebsiella pneumoniae]; >gb|AY847146|83-958|CTX-M-50 [Klebsiella pneumoniae]; >gb|EF418608|10-885|CTX-M-65 [Escherichia coli]; >gb|EF581888|1-876|CTX-M-67 [Escherichia coli]; >gb|FJ214366|1-876|CTX-M-83 [Salmonella enterica subsp. enterica serovar Derby]; >gb|FJ214367|1-876|CTX-M-84 [Salmonella enterica subsp. enterica serovar Derby]; >gb|FJ214369|1-876|CTX-M-86 [Salmonella enterica subsp. enterica serovar Agona]; >gb|HQ166709|1-876|CTX-M-93 [Escherichia coli]; >gb|HM755448|245-1120|CTX-M-98 [Escherichia coli]; >gb|HM803271|1-876|CTX-M-99 [Klebsiella pneumoniae]; >gb|HQ398215|245-1120|CTX-M-102 [Escherichia coli]; >gb|HQ833652|236-1111|CTX-M-104 [Escherichia coli]; >gb|HQ833651|245-1120|CTX-M-105 [Escherichia coli]; >gb|JF274243|1-876|CTX-M-111 [Shigella sp. SH202]; >gb|JF274246|1-876|CTX-M-112 [Shigella sp. SH257]; >gb|JF274247|1-876|CTX-M-113 [Shigella sp. SH284]; >gb|JN790862|245-1120|CTX-M-121 [Escherichia coli]; >gb|JN790863|233-1108|CTX-M-122 [Escherichia coli]; >gb|JQ724542|175-1050|CTX-M-125 [Enterobacter cloacae]; >gb|JX017364|239-1114|CTX-M-129 [Escherichia coli]; >gb|JX896165|1-876|CTX-M-134 [Escherichia coli]; >gb|KF513180|1-876|CTX-M-147 [Klebsiella pneumoniae]; >gb|KJ020574|245-1120|CTX-M-148 [Escherichia coli]; >gb|FJ214368|1-876|CTX-M-85 [Salmonella enterica subsp. enterica serovar Albany]; >gb|AB976602.1|136-1011|CTX-M-159 [Klebsiella pneumoniae]; >gb|EU545409|81-956|CTX-M-87 [Escherichia coli]; >gb|FJ907381|1-876|CTX-M-90 [Salmonella sp. YLD3]; >gb|AY847147|82-957|CTX-M-46 [Klebsiella pneumoniae]; >gb|AB703103|1-876|CTX-M-126 [Escherichia coli]; >gb|KP128034.1|1-876|CTX-M-161 [Escherichia coli]; >gb|HQ913565|1-870|CTX-M-106 [Escherichia coli]; >gb|AY033516|2836-3711|CTX-M-17 [Klebsiella pneumoniae]; >gb|JX017365|245-1120|CTX-M-130 [Escherichia coli]; >gb|JF274242|1-877|CTX-M-110 [Shigella sp. SH165]; >gb|AF252623|1-876|CTX-M-13 [Klebsiella pneumoniae]; >gb|EU136031|1-876|CTX-M-81 [Klebsiella pneumoniae]; >gb|AF174129|6336-7211|CTX-M-9 [Escherichia coli]; >gb|AY029068|1-876|CTX-M-16 [Escherichia coli]; >gb|DQ211987|1-876|CTX-M-51 [Escherichia coli]; >gb|AJ416346|557-1432|CTX-M-21 [Escherichia coli]; >gb|D89862|112-981|CTX-M-45 [Escherichia coli]; >gb|AB900900|1-876|CTX-M-137 [Escherichia coli].

***bla*OXA Gene:**

OXA 23 *like* Subgroup:

>gb|AY795964|1-822|OXA-23 [Acinetobacter baumannii]; >gb|AF201828.2|116-937|OXA-27 [Acinetobacter baumannii]; >gb|HM488986|1-822|OXA-165 [Acinetobacter baumannii]; >gb|HM488987|1-822|OXA-166 [Acinetobacter baumannii]; >gb|HM488988|1-822|OXA-167 [Acinetobacter baumannii]; >gb|HM488989|1-822|OXA-168 [Acinetobacter baumannii]; >gb|HM488990|1-822|OXA-169 [Acinetobacter baumannii]; >gb|HM488991|1-822|OXA-170 [Acinetobacter baumannii]; >gb|HM488992|1-822|OXA-171 [Acinetobacter baumannii]; >gb|JN638887|1-822|OXA-225 [Acinetobacter baumannii]; >gb|JQ837239|1-822|OXA-239 [Acinetobacter sp. enrichment culture clone 8407]; >gb|KP050485|1-822|OXA-366 [Acinetobacter baumannii]; >gb|KM087842|1-822|OXA-398 [Acinetobacter baumannii]; >gb|AY288523|1-825|OXA-49 [Acinetobacter baumannii]; >gb|EU571228|823-1644|OXA-133 [Acinetobacter radioresistens]; >gb|APQF01000011.1|291558-292379|OXA-103 [Acinetobacter radioresistens DSM 6976 = NBRC 102413 = CIP 103788]; >gb|FJ194494|172-996|OXA-146 [Acinetobacter baumannii]; >gb|AY762325|116-937|OXA-73 [Klebsiella pneumoniae]; >gb|KM433671|1-822|OXA-422 [Acinetobacter baumannii]; >gb|KM433672|1-822|OXA-423 [Acinetobacter baumannii]; >gb|KP144324|1-822|OXA-435 [Acinetobacter baumannii]; >gb|KP727574.1|1-822|OXA-440 [Acinetobacter baumannii]; >gb|KP264124.1|77-898|OXA-482 [Acinetobacter baumannii]; >gb|KP264125.1|84-905|OXA-483 [Acinetobacter baumannii].

OXA 24/40 *like* Subgroup:

>gb|AF509241|1-828|OXA-24 [Acinetobacter baumannii]; >gb|AF201826|22-849|OXA-25 [Acinetobacter baumannii]; >gb|AF201827|22-849|OXA-26 [Acinetobacter baumannii]; >gb|AM991978|1-828|OXA-139 [Acinetobacter baumannii]; >gb|JQ838185|1-828|OXA-207 [Acinetobacter pittii]; >gb|GU199038|1196-2023|OXA-160 [Acinetobacter baumannii]; >gb|GU199039.2|1206-2033|OXA-72 [Acinetobacter baumannii]; >gb|KP410856.1|1-828|OXA-437 [Acinetobacter baumannii].

OXA 48 *like* Subgroup:

>gb|KR401105.1|1-798|OXA-484 [Klebsiella pneumoniae]; >gb|JX423831|2677-3474|OXA-232 [Escherichia coli]; >gb|JN205800|4141-4938|OXA-181 [Klebsiella pneumoniae]; >gb|KP264119.1|1-798|OXA-416 [Shewanella xiamenensis]; >gb|KP410734.1|1-792|OXA-438 [Escherichia coli]; >gb|KP727573.1|1-786|OXA-439 [Escherichia coli]; >gb|HQ700343|1-786|OXA-163 [Enterobacter cloacae]; >gb|JX893517|1-786|OXA-247 [Klebsiella pneumoniae]; >gb|JQ809466|5375-6172|OXA-204 [Klebsiella pneumoniae]; >gb|CP022089.2|2724286-2725083|OXA-252 [Shewanella sp. FDAARGOS_354]; >gb|JN704570|4039-4836|OXA-199 [Shewanella xiamenensis]; >gb|JX438001|1-798|OXA-245 [Klebsiella pneumoniae]; >gb|AY236073|2188-2985|OXA-48 [Klebsiella pneumoniae]; >gb|HM015773|2127-2924|OXA-162 [Klebsiella pneumoniae]; >gb|JX438000|1-798|OXA-244 [Klebsiella pneumoniae]; >gb|KF900153|1-798|OXA-370 [Enterobacter sp. 87F-2]; >NG_055490.1 Klebsiella pneumoniae 1210 pKp1210 blaOXA gene for OXA-48 family class D beta-lactamase OXA-519, complete CDS.

OXA 51 *like* Subgroup:

>gb|KF048915.1|1-825|OXA-342 [Acinetobacter baumannii]; >gb|HQ425493|1-822|OXA-195 [Acinetobacter nosocomialis]; >gb|EU670845|1595-2419|OXA-138 [Acinetobacter nosocomialis]; >gb|HQ425492|1-822|OXA-194 [Acinetobacter nosocomialis]; >gb|HQ425494|1-822|OXA-196 [Acinetobacter nosocomialis]; >gb|HQ425495|1-822|OXA-197 [Acinetobacter nosocomialis]; >gb|HM113561|1-825|OXA-175 [Acinetobacter baumannii]; >gb|HM113562|1-825|OXA-176 [Acinetobacter baumannii]; >gb|HM113563|1-825|OXA-177 [Acinetobacter baumannii]; >gb|EU019536|1-825|OXA-82 [Acinetobacter baumannii]; >gb|HQ734812|1-825|OXA-201 [Acinetobacter baumannii]; >gb|HM113564|1-825|OXA-178 [Acinetobacter baumannii]; >gb|EU029998|576-1400|OXA-115 [Acinetobacter baumannii]; >gb|EU019535|1-825|OXA-80 [Acinetobacter baumannii]; >gb|KF986256.1|41-865|OXA-375 [Acinetobacter baumannii]; >gb|DQ519089|9-833|OXA-95 [Acinetobacter baumannii]; >gb|NWUK01000007.1|12140-12964|OXA-343 [Acinetobacter baumannii]; >gb|HE963770|1-825|OXA-249 [Acinetobacter baumannii]; >gb|EF650034|1-825|OXA-108 [Acinetobacter baumannii]; >gb|EU255295.1|1-825|OXA-126 [Acinetobacter baumannii]; >gb|EU547445|1-825|OXA-130 [Acinetobacter baumannii]; >gb|AM231719|1-825|OXA-90 [Acinetobacter baumannii]; >gb|HQ734811|1-825|OXA-200 [Acinetobacter baumannii]; >gb|KF057032|1-825|OXA-315 [Acinetobacter baumannii]; >gb|KF057033|1-825|OXA-316 [Acinetobacter baumannii]; >gb|DQ519088|9-833|OXA-94 [Acinetobacter baumannii]; >gb|KF048918.1|1-825|OXA-345 [Acinetobacter baumannii]; >gb|DQ392963|9-833|OXA-88 [Acinetobacter baumannii]; >gb|AY750908|1-825|OXA-65 [Acinetobacter baumannii]; >gb|EU547446|1-825|OXA-131 [Acinetobacter baumannii]; >gb|KR872296.1|1-825|OXA-480 [Acinetobacter baumannii]; >gb|KM588353|1-825|OXA-425 [Acinetobacter baumannii]; >gb|APOR01000009.1|341737-342561|OXA-260 [Acinetobacter baumannii NIPH 1362]; >gb|AB781687|1-825|OXA-254 [Acinetobacter baumannii]; >gb|HQ734813|1-825|OXA-202 [Acinetobacter baumannii]; >gb|HM113558|1-825|OXA-172 [Acinetobacter baumannii]; >gb|HM113559|1-825|OXA-173 [Acinetobacter baumannii]; >gb|HM113560|1-825|OXA-174 [Acinetobacter baumannii]; >gb|EF650035|1-825|OXA-109 [Acinetobacter baumannii]; >gb|EU019534|1-825|OXA-79 [Acinetobacter baumannii]; >gb|DQ309277|1-825|OXA-83 [Acinetobacter baumannii]; >gb|DQ309276|1-825|OXA-84 [Acinetobacter baumannii]; >gb|AY949203|1-825|OXA-76 [Acinetobacter baumannii]; >gb|KF048907.1|1-825|OXA-336 [Acinetobacter baumannii]; >gb|NG_050607.1|101-925|OXA-234 [Acinetobacter baumannii]; >gb|AB634250|1-825|OXA-206 [Acinetobacter baumannii]; >gb|EU255296.1|1-825|OXA-127 [Acinetobacter baumannii]; >gb|EF016356.1|1-825|OXA-66 [Acinetobacter baumannii]; >gb|APQY01000006.1|416599-417423|OXA-263 [Acinetobacter baumannii NIPH 329]; >gb|KJ584914.1|1-825|OXA-414 [Acinetobacter baumannii]; >gb|KF057031|1-825|OXA-314 [Acinetobacter baumannii]; >gb|JX025021|1-825|OXA-241 [Acinetobacter baumannii]; >gb|KF057027|1-825|OXA-121 [Acinetobacter baumannii]; >gb|KF057029|1-825|OXA-312 [Acinetobacter baumannii]; >gb|KF057030|1-825|OXA-313 [Acinetobacter baumannii]; >gb|KF986255|17-841|OXA-374 [Acinetobacter baumannii]; >gb|AY750913|1-825|OXA-71 [Acinetobacter baumannii]; >gb|AM231720|1-825|OXA-100 [Acinetobacter baumannii]; >gb|PYSX01000023.1|160179-161003|OXA-337 [Acinetobacter baumannii]; >gb|KJ135342.1|49-873|OXA-390 [Acinetobacter baumannii]; >gb|KM979379.1|15-839|OXA-432 [Acinetobacter baumannii]; >gb|KJ135344|15-839|OXA-381 [Acinetobacter baumannii]; >gb|KJ135345|15-839|OXA-382 [Acinetobacter baumannii]; >gb|KF048911.1|1-825|OXA-339 [Acinetobacter baumannii]; >gb|NGCN01000023.1|65771-66595|OXA-340 [Acinetobacter baumannii]; >gb|EF650032|1-825|OXA-106 [Acinetobacter baumannii]; >gb|NG_049717.1|14-838|OXA-430 [Acinetobacter baumannii]; >gb|CU468230.2|1959578-1960402|OXA-75 [Acinetobacter baumannii SDF]; >gb|KF986263.1|91-915|OXA-384 [Acinetobacter baumannii]; >gb|KJ584917.1|1-825|OXA-408 [Acinetobacter baumannii]; >gb|JX025022|1-825|OXA-242 [Acinetobacter baumannii]; >gb|DQ445683|1-825|OXA-89 [Acinetobacter baumannii]; >gb|EU220744|1-786|OXA-116 [Acinetobacter baumannii]; >gb|GQ853680|1-825|OXA-149 [Acinetobacter baumannii]; >gb|KF057034|1-825|OXA-317 [Acinetobacter baumannii]; >gb|GQ853681|1-825|OXA-150 [Acinetobacter baumannii]; >gb|KP844571.1|1-825|OXA-442 [Acinetobacter baumannii]; >gb|KM588354|1-825|OXA-426 [Acinetobacter baumannii]; >gb|FJ872530|1-825|OXA-144 [Acinetobacter baumannii]; >gb|AY750910|1-825|OXA-68 [Acinetobacter baumannii]; >gb|EU375515|1-825|OXA-128 [Acinetobacter baumannii]; >gb|KF986254|35-859|OXA-386 [Acinetobacter baumannii]; >gb|GQ423625.1|1-825|OXA-117 [Acinetobacter baumannii]; >gb|JX865394.1|1-825|OXA-441 [Acinetobacter baumannii]; >gb|JN603240|1-825|OXA-217 [Acinetobacter baumannii]; >gb|AY862132|1-825|OXA-78 [Acinetobacter baumannii]; >gb|KM979378.1|15-839|OXA-431 [Acinetobacter baumannii]; >gb|DQ519086|1199-2023|OXA-91 [Acinetobacter baumannii]; >gb|AY949202|1-825|OXA-77 [Acinetobacter baumannii]; >gb|AM279652|1-825|OXA-98 [Acinetobacter baumannii]; >gb|KF986259.1|17-841|OXA-378 [Acinetobacter baumannii]; >gb|KJ135343|15-839|OXA-388 [Acinetobacter baumannii]; >gb|KF048913.1|1-825|OXA-341 [Acinetobacter baumannii]; >gb|KJ780078.1|1-825|OXA-400 [Acinetobacter baumannii]; >gb|KF986262.1|28-852|OXA-383 [Acinetobacter baumannii]; >gb|KJ584922.1|1-825|OXA-413 [Acinetobacter baumannii]; >gb|JN248564|1-825|OXA-223 [Acinetobacter baumannii]; >gb|KM979376.1|21-845|OXA-429 [Acinetobacter baumannii]; >gb|KM588352|1-825|OXA-424 [Acinetobacter baumannii]; >gb|DQ519087|39-863|OXA-93 [Acinetobacter baumannii]; >gb|KF986253|16-840|OXA-385 [Acinetobacter baumannii]; >gb|KJ780076.1|1-825|OXA-401 [Acinetobacter baumannii]; >gb|EF653400|435-1259|OXA-113 [Acinetobacter baumannii]; >gb|KF048909|1-825|OXA-338 [Acinetobacter baumannii]; >gb|HQ998857|1-825|OXA-203 [Acinetobacter baumannii]; >gb|KF048917.1|1-825|OXA-344 [Acinetobacter baumannii]; >gb|EF650037|1-825|OXA-111 [Acinetobacter baumannii]; >gb|KJ584916.1|1-825|OXA-407 [Acinetobacter baumannii]; >gb|HM570035|1-825|OXA-179 [Acinetobacter baumannii]; >gb|KF305666.1|1-825|OXA-259 [Acinetobacter baumannii]; >gb|HM570036|1-825|OXA-180 [Acinetobacter baumannii]; >gb|FR865168|1-825|OXA-216 [Acinetobacter baumannii]; >gb|AY750907|1-825|OXA-64 [Acinetobacter baumannii]; >gb|KF885217|1-825|OXA-365 [Acinetobacter baumannii]; >gb|KJ920338.1|1-825|OXA-404 [Acinetobacter baumannii]; >gb|APRA01000005.1|465154-465978|OXA-262 [Acinetobacter baumannii NIPH 67]; >gb|DQ888718|1-825|OXA-99 [Acinetobacter baumannii]; >gb|KJ584920.1|1-825|OXA-411 [Acinetobacter baumannii]; >gb|KF986257|21-845|OXA-376 [Acinetobacter baumannii]; >gb|KF986258|15-839|OXA-377 [Acinetobacter baumannii]; >gb|EU255291.1|1-825|OXA-122 [Acinetobacter baumannii]; >gb|JN215211|1-825|OXA-219 [Acinetobacter baumannii]; >gb|AJ309734|1-825|OXA-51 [Acinetobacter baumannii]; >gb|EU547447|1-825|OXA-132 [Acinetobacter baumannii]; >gb|KJ584918.1|1-828|OXA-409 [Acinetobacter baumannii]; >gb|KJ584915.1|1-825|OXA-406 [Acinetobacter baumannii]; >gb|KF986260|21-845|OXA-379 [Acinetobacter baumannii]; >gb|EU255292.1|1-825|OXA-123 [Acinetobacter baumannii]; >gb|KJ584921.1|1-825|OXA-412 [Acinetobacter baumannii]; >gb|KJ427797|1-825|OXA-391 [Acinetobacter baumannii]; >gb|GQ853679|1-825|OXA-148 [Acinetobacter baumannii]; >gb|AY750912|1-825|OXA-70 [Acinetobacter baumannii]; >gb|EU255294.1|1-825|OXA-125 [Acinetobacter baumannii]; >gb|KJ780077.1|1-825|OXA-402 [Acinetobacter baumannii]; >gb|KF048919.1|1-825|OXA-346 [Acinetobacter baumannii]; >gb|HE963771|1-825|OXA-250 [Acinetobacter baumannii]; >gb|HE963769|1-825|OXA-248 [Acinetobacter baumannii]; >gb|EF650038|1-825|OXA-112 [Acinetobacter baumannii]; >gb|EF650036|1-825|OXA-110 [Acinetobacter baumannii]; >gb|EF650033|1-825|OXA-107 [Acinetobacter baumannii A424]; >gb|DQ335566|1-825|OXA-92 [Acinetobacter baumannii]; >gb|KJ920337.1|1-825|OXA-403 [Acinetobacter baumannii]; >gb|AB871653|11871-12695|OXA-371 [Acinetobacter baumannii]; >gb|CU459141.1|2175317-2176141|OXA-69 [Acinetobacter baumannii AYE]; >gb|APQV01000009.1|266253-267077|OXA-261 [Acinetobacter baumannii NIPH 201]; >gb|KM979380.1|26-850|OXA-433 [Acinetobacter baumannii]; >gb|EU255293.1|1-825|OXA-124 [Acinetobacter baumannii]; >gb|FR853176|1-825|OXA-208 [Acinetobacter baumannii]; >gb|DQ491200|1-825|OXA-67 [Acinetobacter baumannii]; >gb|DQ149247|1-825|OXA-86 [Acinetobacter baumannii]; >gb|DQ348075|1-825|OXA-87 [Acinetobacter baumannii]; >gb|KF986261|45-869|OXA-380 [Acinetobacter baumannii]; >gb|HE963768|1-825|OXA-120 [Acinetobacter baumannii].

**Text S2:** Consensus sequences obtained for the gene groups analyzed.

***bla*SHV Gene consensus:**

------NNNNNNNNTNTTCGCCNGNNTATTATCTCCCTGTTAGCCACCCTGCCGCTGGCGGTA

CACGCCAGCCCGCAGCCGCTTGAGCAAATTAAACNAAGCGAAAGCCAGC---------------------------------------TGTCGGGCNGCGTAGGCATGATAGAAATGGATCTGGCCAGCGGCCGCACGCT

GACCGCCTGGCGCGCCGATGAACGCTTTCCCATGATGAGCACCTTTAAAGTAGTGCTCTGCGGCGCAGTGCTGGCGCGGGTGGATGCCGGTGACGAA------NNNNNNNNTNTTCGCCNGN

NTATTATCTCCCTGTTAGCCACCCTGCCGCTGGCGGTACACGCCAGCCCGCAGCCGCTTGAGCAAATTAAACNAAGCGAAAGCCAGC---------------------------------------TGTCGGGCNGCGTA

GGCATGATAGAAATGGATCTGGCCAGCGGCCGCACGCTGACCGCCTGGCGCGCCGATGAACGCTTTCCCATGATGAGCACCTTTAAAGTAGTGCTCTGCGGCGCAGTGCTGGCGCGGGTGGATGCCGGTGACGAA------NNNNNNNNTNTTCGCCNGNNTATTATCTCCCTGTTAGCCAC

CCTGCCGCTGGCGGTACACGCCAGCCCGCAGCCGCTTGAGCAAATTAAACNAAGCGAAAGCCAGC---------------------------------------TGTCGGGCNGCGTAGGCATGATAGAAATGGATCTG

GCCAGCGGCCGCACGCTGACCGCCTGGCGCGCCGATGAACGCTTTCCCATGATGAGCACCTTTAAAGTAGTGCTCTGCGGCGCAGTGCTGGCGCGGGTGGATGCCGGTGACGAA------N

NNNNNNNTNTTCGCCNGNNTATTATCTCCCTGTTAGCCACCCTGCCGCTGGCGGTACACGCCAGCCCGCAGCCGCTTGAGCAAATTAAACNAAGCGAAAGCCAGC---------------------------------------TGTCGGGCNGCGTAGGCATGATAGAAATGGATCTGGCCAGCGGCCGCACGCTGACC

GCCTGGCGCGCCGATGAACGCTTTCCCATGATGAGCACCTTTAAAGTAGTGCTCTGCGGCGCAGTGCTGGCGCGGGTGGATGCCGGTGACGAA

***bla*TEM Gene consensus:**

----------------------------------------------------------------------------------------------------ATGAGTATTNAACATTTNCGTGTCGCCCTTATTCCCTTTTTTGCGGCATTTTGCNTTCCTGTTTTTGCTCACCCAGAAACGCTGGTGAAAGTAAAAGATGCTGAAGATNAGTTGGGTGCACGAGTGGGTTACATCGANCTGGATCTCAACAGCGGTAAGATCCTTGAGAGTTTTCGCCCCGAAGAACGTTTTCCAATGNTGAGCACTTTTAAAGTTCTGCTATGTGGNGCGGTATTATCCCGTGTTGACGCCGGGCAAGAGCAACTCGGTCGCCGCATACACTATTCTCAGAATGACTTG

***bla*NDM gene consensus:**

ATGGAATTGCCCAATATTATGCACCCGGTCGCGAAGCTGAGCACCGCATTAGCCGCTGCATTGATGCTGAGCGGGTGCATGCCCGGTGAAATCCGCCCGACGATTGGCCAGCAAATGGAAACTGGCGACCAA---------------CGGTTTGGCGATCTGGTTTTCCGCCAGCTCGCACCGAATGTCTGGCAG

CACACTTCCTATCTCGACATGCCGNGTTTCGGGGCAGTCGCTTCCAACGGTTTGATCGTCAGGGATGGCGGCCGCGTGCTGNTGGTCGATACCGCCTGGACCNAT

***bla*KPC Gene consensus:**

NNNNNNNNNNNNNGCCNTCTAGTTCTGCTGTCTTGTCTCTCATGGCCGCTGGCTGGCTTTTCTGCCACCGCGCTGACCAACCTCGTCGCGGAACCATTCGCTAAACTCGAACAGGACTTTGGCGGCTCCATCGGTGTGTACGCGATNGATACCGGNTCAGGCGCAACTGTAAGTTACCGCGCTGAGGAGCGCTTCCCACTGTGCAGCTCATTCAAGGGCTTTCTTGCTGCCGCTGTGCTGGCTCGCAGCCAGCAGCAGGCCGGCTTGCTGGNCACACCCATCCGTTACGGCAAAAATGCGCTGGTTCNGNGGTCACCCATCTCGGAAAAATATCTGACAACAGGCATGACGGTNNNGGAGCTGTCCGCGGCCGCCGTGCAATACAGTGATAACGCCGCCGCCAATTTGTTGCTGAAGGAGTTGGGCGGCCCGGCCNNNCTGACGGCCTTCATGCGCTCTATCGGCGATACCACGTTCCGTCTGGACCGCTGGGAGCTGGAGNTGAACTCCGCNATCCCAGGCGATGCGCGCGATACCTCATCGCCGCGCGCCGTGACGGAAAGCTTACAAAAACTGACACTGGGCTCTGCACTGGCTGCGNCGCAGCGGCAGCAGNTTGTTGATTGGCTAAAGGGAAACACGACCGGCAACCACCGCATCCGCGCGGCGGTGCCGGCAGACTGGGCAGTCGGAGACAAAACCGGAACCTGCGGAGNGTATGNNNNNNCAAATGACTATGCCGTCGTCTGGCCCACTGGGCGCGCACCTATTGTGTTGGCCGTCTACACCCGGGCGCCTAACAAGGATGACAAGNACAGCGAGGCCGTCATCGCCGCTGCGGCTAGACTCGCGCTCGAGGGATTGGGCNNNNNNNNNNNNNNN

***bla*GES Gene consensus:**

ATGCGCTTCATTCACGCNCTATTACTGGCAGNGATCGCTCACTCTGCATATGCNTCGGAAAAATTAACCTTCAAGACCGATCTTGAGAAGCTAGAGCGCGAAAAAGCAGCTCAGATCGGTGTTGCGATCGTCGATCCCCAAGGAGAGATCGTCGCGGGCCACCGAANGGCGCAGCGNTTTGCAATGTGCTCAACGTTCAAGTTTCCGCTAGCCGCGCTGGTCTTTGAAAGAATTGACTCAGGCACCGAGCGGGGGGATCGAAAACTTTCATATGGGCCGGACATGATCGTCNAATGGTCTCCTGCCACGGAGCGGTTTCTAGCATCGGGACACATGACGGTTCTCGAGGCAGCGCAAGCNGCGGTGCAGCTTAGCGACAATGGGGCTACTAACCTCTTACTGAGAGAAATTGGCGGACCTGCTGCAATGACGCAGTATTTTCGTAAAATTGGCGACTCTGTGAGTCGGCTAGACCGGAAAGAGCCGGAGATGNNCGACAACACACCTGGCGACCTCAGAGATACAACTACGCCTATTGCTATGGCACGTACTGTGGCNAAAGTCCTCTATGGCGGCGCACTGACGTCCACCTCGACCCACACCATTGAGAGGTGGCTGATCGGAAACCAAACGGGAGACGCGACNCTACGAGCGGGTTTTCCTAAAGATTGGGTTGTTGGAGAGAAAACTGGTACCTGCGCCAACGGGGNCCGGAACGACATTGGTTTTTTTAAAGCCCAGGAGAGAGATTACGCTGTAGCGGTGTATACAACGGCCCCGAAACTATCGGCCGTAGAACGTGACGAATTAGTTGCCTCTGTCGGTCAAGTTATTACACAACTCATCCTGAGCACGGACAAATAG

***bla*CTX-M Gene:**

CTX-M 1.*1like Consensus*:

NNGGTTAAAAAATCACTGCGNCAGTTCACGCTGATGGCGACGGCANCCGTCACGCTGTTGTTAGGAAGTGTGCCGCTGTATGCGCAAACGGCGGACGTACAGCAAAAACTTGCCGAATTAGAGCGGCAGTCGGGAGGCAGACTGGGNGTGGCATTGATTAACACAGCNGATAATTCGCAAATACTTTATCGTGCTGATGAGCGCTTNGCGATGTGCAGCACCAGTAAAGTGATGGCCGNGGCCGCGGTGCTGAAGAAAAGTGAAAGCGAACCGANTCTGTTAAATCAGCGAGTTGAGATCAAAAAATCTGACCTNGTTAACTATAATCCGATTGCGGAAAAGCACGTCAATGGGACGATGTCACTGGCTGAGCTTAGCGCGGCCGCGCTACAGTACAGCGATAACGTGGCGATGAATAAGCTGATTGCTCACGTTGGCGGCCCGGCTAGCGTCACCGCGTTCGCCCGACAGCTGGGAGACGAAACGTTCCGTCTCGACCGTACCGAGNCGACGTTAAACACCGCCATTCCGGGCGATCCGCGTGATACCACTTCACCTCGGGCAATGGCGCAAACTCTGCGGAATCTGACGCTGGGTAAAGCATTGGGCGACAGCCAACGGGCGCAGCTGGTGACATGGATGAAAGGCAATACCACCGGTGCAGCGAGCATTCAGGCNGGACTGCCTGCTTCCTGGGTTGTGGGGGATAAAACCGGCAGCGGTGNCTATGGCACCACCAACGATATCGCGGTGATNTGGCCAAAAGATCGTGCGCCGCTGATTCTGGTCACTTACTTCACCCAGCCTCAACCTAAGGCAGAAAGCCGTCGCGATNTATTAGCGTCGGCGGCTAAAATCGTCACCNANNNNNNNNNN

CTX-M 1.*2like Consensus*:

ATGGTTAAAAAATCACTGCGNCAGTTCACGCTGATGGCGACGGCAACCGTCACGCTGTTNTTAGGAAGTGTGCCGCTGNATGCGCAAACGGNGGACGTACAGCAAAAACTTGCCGAATTAGAGCNGCAGTCGGGAGGNNGNCTGGGTGTGGCATTGATTAACACNGCNGATAATTCGCAAATACTTTATCGTGCNGATGAGCGNTTTNCNATGTGCAGNACCAGTAAAGTNATGGCNGNNGCNGCGGTGCTNAAGNANAGTGAAANNNAANNGNANCTGNTNAATCAGCNNGTNGAGATCAANNNNNCNGANNTNGTTAACTANAATCCGATTGCNGAAAANCACGTCNANGGNACNATGNCNCTGGNNGANCTNAGCGNGGCCGCGNTNCAGTACAGCGANAANNNNGCNATGAANAANNTGATTNCNCANNTNGGNGGCCCGGNNNGCGTNACNGCNTTNGCCCGNNNGNTNGGNGANGANACGTTNCGTCTNGANCGNACNGANNCNACGNTNAANACNGCCATTCCNGGCGANCCGNGNGANACCACNNCNCCNCGGGCNATGGCGCANACNNTGCGNNANCTNACGCTGGGTNANGCNNTGGGNGANANCCANCGGGCGCAGNTGGTGACNTGGNTNAAAGGCAATACNACCGGNGCNGCNAGNATTCNGGCNGGNNTNCCNNCNTCNTGGNNTGTGGGNGATAANACCGGCAGCNGTGNCTATGGNACCACCAACGATATCGCGGTGATNTGGCCAAAAGATCGTGCGCCGCTGATTCTGGTCANTTACTTCACCCAGCCNCAACCTAAGGCAGAAAGCCGTCGCGATGTATTAGCGTCGGCGGCTAAAATCGTCACCNACGGTTTGTAN

CTX-M 2*like* Consensus:

ATGATGACNCAGAGCATTCGCCGCNNNATGNTNACNGTGATGGCGACNCTNCCCCTGCTNTTTAGCAGCGCNACNCTGCANGCGCAGNCGAACAGCGTGCANCAGCAGCTGGAAGCNCTGGANAAAAGNNNNGGNGGNCGNCTNGGCGTNGCGCTGATTAACACCGCNGATAANNNNCAGATTCTNTANNNNGCNGATGANCGNTTTGCGATGTGCAGNACCAGNAANGTGATGGCGGCNGCGGCGGTGCTNAAACAGAGCGANAGCGATAANCANCTGCTNAANCAGCGCGTNGAAATNANNNNNAGCGANCTGGTNAACTANAANCCNATTGCNGANAAACANGTNAACGGCACNATGACNCTGGCNGANCTNGGCGCNGNNGCNCTGCAGTATAGCGANAANACNGCNATGAANAANCTGATTGCNCATCTGGGNGGNCCNGATAAAGTGACNGCGTTTGCNCGCNNNNTGGGNGATGANACCTTNCGNCTGGANNGNACCGANNCCACNCTNAANANCGCNATTCCNGGCGANCCGCGNGATACCACCACNCCGCTNGCGATGGCGCAGACCCTGAAAAANCTGACNCTGGGNAAAGCGCTGGCGGAAACNCAGCGNGCNCAGNTGGTGACNTGGCTNAANGGCAANACNACCGGNAGCGCGAGCATTCNNGCGGGNCTGCCGAAANNNTGGGNNGTGGGCGATAAAACCGGCAGCGGNGNTTATGGCACCACCAACGATATNGCGNTNATNTGGCCGGAAAACCANGCNCCGCTGGTNCTGGTGACCTANTTTACCCANCCGGANCAGAANGCGGAAAGNCGNCGNGATNTTCTGGCNGCGGCGGCGAAAATNGTNACCCANGGNTTNNNN

CTX-M *8like Consensus*:

NNNATGAGANANNGCGTNANGCGGNNGATNNTAATGACNACNGCCTGTNTTTCGCTGNTGNTGGNNAGTGNGCCGCTGTNTGCNCANGCGAACGANNTTCANCANAAGCTNGCGGCGCTGGAGAAAAGCAGCGGGGGNCGNNTGGGNGTGGCGNTGATTNACACCGCCGATAACNCNCAGACGCTCTACCGCGCCGANGAGCGNTTTGCNATGTGCAGCACCAGTAANGTGATGGCNGNNGCGGCNGTGCTNAAGCAAAGTGAAACGCAAAANNNNNTNNTGAGTCAGNNGGTTGANATTAANNCNTCNGACNTGNTTAACTACAANCCNATNNCNGAAAANCACGTCAANGGCACGATGACNNTNGNGGANNTGANCGCNGCGGCGNTNCAGTACAGCGANAATACNGCCATGAANAAGCTGATTGCCCATCTNGGGGGGCCGGNTAAAGTGACGGCNTTTGCNCGNNNGATTGGNGATNACACNTTCCGGCTCGATCGTACNGAGCCGACGCTCAACACCGCGATCCCCGGCGACCCGCGCGATACCACCACGCCNTTAGCGATGGCGCAGNCNCTNCGCNATCTNACNTTGGGCANTGCCNTNGGTGANACTCAGCGTGCGCANCTGGTNANGTGGCTGAAAGGCAANACCACCGGNGCTGCCAGCATTCNGGCNGGGCTACCCACATCGTGGGTTGTCGGGGATAAAACCGGCAGCGGNGNTTATGGTACGACGAATGANATCGCNGTNATNTGGCCGGAAGGNCGNGCGCCGCTNNTTCTGGTNACTTACTTCACCCANNCNGAGCNGAAGGCAGANANNCGTCGTGACGTNCTCGCNGCTGCCGCNANAATNGTCACCGACGGTTATTAN

CTX-M *9like Consensus*:

ATGGTGACAAAGAGAGTGCAACGGATGATGTTCGCGGCGGCGGCGTGCATTCCGCTGCTGCTGGGCAGCGCGCCGCTTTATGCGCAGACGAGTGCGGTGCAGCAAAAGCTGGCGGCGCTGGAGAAAAGCAGCGGAGGGCGGCTGGGCGTCNCGCTCATCGATACCGCAGATAATACGCAGGTGCTTTATCGCGGTGATGAACGCTTTCCAATGTGCAGTACCAGTAAAGTTATGGCGGNCGCGGCGGTGCTTAAGCAGAGTGAAACGCAAAAGCAGCTGCTTAATCAGCCTGTCGAGATCAAGCCTGCCGATCTGGTTAACTACAATCCGATTGCCGAAAAACACGTCAACGGCACAATGACGCTGGCAGANCTGAGCGCGGCCGCGTTGCAGTACAGCGACAATACCGCCATGAACAAATTGATTGCCCAGCTCGGTGGCCCGGGAGGCGTGACGGCTTTTGCCCGCGCGATCGGCGATGAGACGTTTCGTCTGGATCGCACTGAANCTACGCTGAATACCGCCATTCCCGGCGACCCGAGAGACACCACCACGCCGCGGGCGATGGCNCAGACGTTGCGTCAGCTTACGCTGGGTCATGCGCTGGGCGAAACCCAGCGGGCGCAGTTGGTGACGTGGCTCAAAGGCAATACGACCGGCGCAGCCAGCATTCGGGCCGGCTTACCGACGTCGTGGACTGNNGGTGATAAGACCGGCAGCGGCGNCTACGGCACCACCAATGATATTGCGGTGATCTGGCCGCAGGGTCGTGCGCCGCTGGTTCTGGTGACCTATTTTACCCAGCCGCAACAGAACGCAGAGNGCCGCCGCGATGTGCTGGCTTCAGCGGCGAGAATCATCGCCGAAGGGNTGTNA

***bla*OXA Gene:**

OXA Consensus Subgroup 23 *like*:

ATGAATAAATATTTTACTTGCTATGTGGTTGCTTCTCTTTTTCTTTCTGGTTGTACGGTTCAGCATAATTTAATAAATGAAACCCNGAGTCAGATTGTTCAAGGACATAATCAGGTGATTCATCAATACTTTGATGAAAAAAACACCTCAGGTGTGCTGGTTATTCAAACAGATAAAAAAATTAATCTATATGGTAATGCTCTAAGCCGCGCAAATACAGAATATGTGCCAGCCTCTACATTTAAAATGTTGAATGCCCTGATCGGATTGGAGAACCAGAAAACGGATATTAATGAAATATTTAAATGGAAGGGCGAGAAAAGGTCATTTACCGCTTGGGAAAAAGACATGACACTAGGAGAAGCCATGAAGCTTTCTGCAGTCCCAGTCTATCAGGAACTTGCNNGACGTATCGGTCTTGATCTCATGCAAAAAGAAGTANAACGTATTGNTTTCGGTAATGCTGAAATTGGACAGCAGGTTGANAATTTCTGGTTGNTAGGNCCATTAAAGGTNACGCCTATTCAAGAGGTAGAGTTTGTTTCNCAATTNGCACATACACAGCTTCCATTTAGTGAAAAAGTGCAGGCTAATGTAAAAAATATGCTNCTTNTAGAAGAGAGTAATGGCTACAANATTTTTGGAAAGACTGGTTGG---GCAATGGATATAAAANCACA

AGTGGGCTGGTTGNCCGGCTGGGTTGAGCAGCCAGATGGAAAAATTGTCGCTTTTGCATTAAANATGGAAATGCGGTCAGAAATGCCNGCATCTATACGTAATGAATTATTGATGAAATCATTAAAACAGCTGAATATTATTTAA

OXA Consensus Subgroup 24/40 *like*:

ATGAAAAAATTTATACTTCCTATATTCAGCATTTCTATTCTAGTTTCTCTCAGTGCATGTTCATCTATTAAAACTAAATCTGAAGATAATTTTCATATTTCTTCTCAGCAACATGAAAAAGCTATTAAAAGCTATTTTGATGAAGCTCAAACACAGGGTGTAATTATTATTAAAGAGGGTAAAAATCTTAGCACCTATGGTAATGCTCTTGCACGAGCAAATAAAGAATATGTCCCTGCATCAACATTTAAGATGCTAANTGCTTTAATCGGGCTAGAAAATCATAAAGCAACAACAAATGAGATTTTCAAATGGGATGGTAAAAAAAGAACTTATCCTATGTGGGAGAAAGATATGACTTTAGGTGAGGCAATGGCATTGTCAGCAGTTCCAGTATATCAAGAGCTTGCAAGACGGACTGGCCTAGAGCTAATGCAGAAAGAAGTAAAGCGGGTTAATTTTGGAAATACAAATATTGGAACACAGGTCGATAATTTTTGGTTAGTTGGCCCCCTTAAAATTACACCAGTACAAGAAGTTAATTTTGCCGATGACCTTGCACATAACCGATTACCTTTTAAATTAGAAACTCAAGAAGAAGTTNAAAAAATGCTTCTAATTAAAGAAGTAAATGGTAGTAAGATTTATGCAAAAAGTGGATGGGNAATGGNTGTTACTNCACAGGTAGGTTGGTTGACTGGTTGGGTGGAGCAAGCTAATGGAAAAAAAATCNCCTTTTCGCTCAACTTAGAAATGAAAGAAGGAATGNCTGGTTCTATTCGTAATGAAATTACTTATAAGTNGCTAGAAAATCTTGGAATCATTTAA

OXA Consensus Subgroup 48 *like*:

ATGCGTGTATTAGCCTTATCGGCTGTGTTTTTGGTGGCATCGATTATCGGAATGCCNGCGGTAGCAAAGGAATGGCAAGAAAACAAAAGTTGGAATGCTCACTTTACTGAANATAAATCACAGGGCGTAGTTGNGCTCTGGAATGAGAATAAGCAGCAAGGATTTACCAATAATCTTAAACGGGCGAACCAAGCATTTTTACCCGCATCTACCTTTAAAATTCCCAATAGCTTGATCGCCCTCGATTTGGGCGTGGTTAAGGATGAACACCAAGTCTTTAAGTGGGATGGACANNNGCGNGATATCGCCNCTTGGAATCGNGANCATNANNTAATNACCGCGATGAANTANTCNGTTNTGCCTGTTNATCAANANTTTGCCCGCCAAATTGGNGAGGCACGTATGAGNAANATGCTNCANGCNTTCGATTATGGNAATGAGGANATNTCGGGCAATNTAGNNANTTTNTGGCTNGANGGTGGNATTCGNATTTCGGCNACNNAGCAAATCNNNTTTTTANGNAAGCTGTATCACAANAAGNTNCACGTNTCNGAGCGNAGNCAGCGNATNGTNAAACAAGCCATGCTNACNGANGCNAATGNNGACTATATTATTCGGGCTAAAACNGGNNNNNNNNNNNNNNNCNANNCTAAGATTGGCTGGTGGGTNGGTTGGGTTGANCTNGATGATAATGTGTGGTTTTTTGCGATGAATATGGATATGCCCACATCGGATGGTTTAGGGCTGCGCCAAGCCATCACAAAAGAAGTGCTCAAACAGGANAAAATTATTCCCTAG

OXA Consensus Subgroup 51 *like*:

ATGAACATTNAANCNCTCTTACTTATAACAAGCGCTATTTTTATTTCAGCC---TGCTCACCTT

ATATAGTGNCTGCTAATCCAAATCACAGNGCTTCAAAATCTGATNNNAAAGCAGAGAAAATTAAAAATTTATTTAACGAAGNACACACTACGGGTGTNTTAGTTATCCANCAAGGNCAAACTCAACAAAGCTATGGTAATGATCTTGCTCGTGCTTCGACCGAGTATGTACCTGCTTCGACCTTCAAAATGCTTAATGCTTTGATCGGCCTTGAGCACCATAAGNCAACCACNACAGAAGTATTTAANTGGNANGGNNAAAAAAGGNTNTTCCCAGAATGGGAAAAGNACATGACCCTAGGCGANGCNATGAAAGCTTCCGCTATTCNNGTTTATCAAGATTTAGCTCGTCGTATTGGACTTGANCTCATGTCTAANGAAGTGAAGCGTGTTGGTTATGGCAATGCAGATATCGGTACCCAAGTCGATAATTTTTGGNTNGTGGGTCCTNTAAAAATTACNCCTCAGCAAGAGGCACANTTTGCTTACAAGCTAGCTAATAAAACGCTTCCATTTAGCCNAAAAGTCCAAGATGAAGTGCAATCCATGNTATTCATAGAAGAAAAGAATGGAAANAAAATATACGCAAAAAGTGGTTGGGGATGGGATGTANACCCACAAGTAGGCTGGTTAACTGGATGGGTTGTTCAGCCTCAAGGNAATATTGTAGCNTTCTCCCTTAACTTAGAAATGAAAAAAGGAATACCTAGCTCTGTTCGAAAAGAGATTACTTATAAAAGNTTAGAACAATTAGGTATTTTATAG


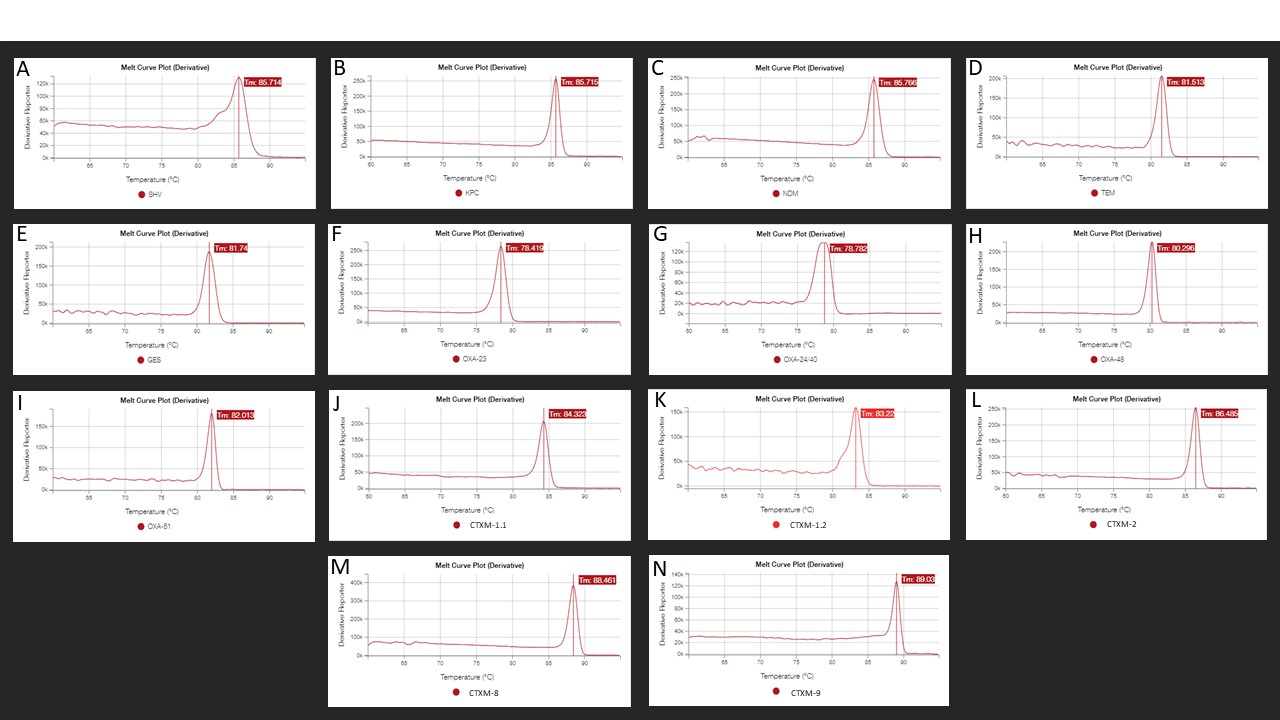


**Figure S1:** Primer’s specificity evaluation through Melting curve: (A) SHV, (B) KPC, (C) NDM, (D) TEM, (E) GES, (F) OXA-23like, (G) OXA-24/40like, (H) OXA-48like, (I) OXA-51like, (J) CTX-M 1.1like, (K) CTX-M 1.2like, (L) CTX-M 2like, (M) CTX-M 8like e (N) CTX-M 9like.
